# Healthcare providers’ use and acceptance of translation technologies in multilingual healthcare settings: a cross-sectional study

**DOI:** 10.1186/s12939-026-03085-6

**Published:** 2026-10-21

**Authors:** Annika Kreienbrinck, Saskia Hanft-Robert, Muhammed-Talha Topçu, Ovidiu Oltean, Alina-Ioana Forray, Răzvan Mircea Cherecheș, Mike Mösko

**Affiliations:** 1 Department of Medical Psychology, University Medical Center Hamburg-Eppendorf, Martinistraße 52, Building West 26, 20246 Hamburg, Germany; 2 Department of Applied Human Sciences, Magdeburg-Stendal University of Applied Sciences, Stendal, Germany; 3 Faculty of Political, Administrative and Communication Sciences, Babeș-Bolyai University, Cluj-Napoca, Romania; 4 Department of Community Medicine, Discipline of Public Health and Management, Iuliu Hațieganu University of Medicine and Pharmacy, Cluj-Napoca, Romania; 5 Division of Health Systems and Public Health, Faculty of Medicine and Health Sciences, Stellenbosch University, Stellenbosch, South Africa; 6 Department of Public Health, Babeș-Bolyai University, Cluj-Napoca, Romania; 7 Department of Psychiatry and Psychotherapy, Central Institute of Mental Health (CIMH), Medical Faculty Mannheim, University of Heidelberg, Mannheim, Germany

**Keywords:** Translation technologies, Language barriers, Mental healthcare, Technology acceptance model, Health professionals, Multilingual healthcare, Cross-sectional survey, Equity in healthcare, Digital health, Diversity

## Abstract

**Background:**

Language barriers remain a major challenge to equitable healthcare delivery. While qualified interpreters are considered the preferred standard, their availability is often limited. Translation technologies are therefore increasingly used in practice. However, quantitative evidence on how healthcare providers use these tools, how they evaluate them, and which factors are associated with their acceptance remains limited.

**Methods:**

A cross-sectional online survey was conducted among healthcare and psychosocial service providers across a range of clinical settings. Inferential analyses were restricted to the German sample (*n* = 258), with Romanian data (*n* = 185) used for exploratory descriptive comparison. The survey assessed experiences with language barriers, communication strategies, and acceptance of translation technologies based on the Technology Acceptance Model. Descriptive analyses characterised use patterns, and multivariable ordinary least squares regression with HC3-robust inference examined factors associated with behavioural intention to use translation technologies.

**Results:**

A total of 443 healthcare providers were included. Translation technologies were used by 65.0% of providers in the previous three months, often in the absence of formal organisational support, training, or guidelines. Use varied descriptively across country samples and professional groups, with higher use observed in the German sample and among medical-technical and nursing staff. Acceptance was moderately positive, with perceived usefulness showing the strongest controlled association with behavioural intention to use translation technologies (β = 0.44, *p* < 0.001). Organisational factors and contextual concerns were not independently associated with behavioural intention. Providers reported concerns regarding accuracy and data privacy, and more cautious use in contexts requiring nuanced, relational communication.

**Conclusions:**

Translation technologies appear to function as pragmatic, ad hoc solutions to communication challenges in multilingual healthcare. While these tools may facilitate basic communication, their limitations in complex interactions and the absence of governance frameworks raise important considerations for their safe and equitable implementation. Rather than replacing professional interpreting services, translation technologies may be best understood as complementary resources within a broader communication strategy. Their contribution to equitable care will depend on how they are implemented to support meaningful communication and address the needs of service users facing language barriers.

**Supplementary Information:**

The online version contains supplementary material available at https://doi.org/10.1186/s12939-026-03085-6.

## Introduction

### Background

Language barriers between healthcare providers and service users remain a major obstacle to equitable and safe healthcare delivery worldwide. Miscommunication can contribute to diagnostic errors, reduced treatment adherence, lower satisfaction, and premature treatment discontinuation [1–3]. As such, language discordance is not only a communication issue, but a structural determinant of unequal access to and quality of care, particularly affecting individuals with limited proficiency in the dominant languages of healthcare systems [4, 5]. These challenges are particularly pronounced in clinical contexts that rely heavily on verbal exchange, such as psychosocial and mental healthcare [6, 7].

Despite widespread recognition of the importance of effective communication, addressing language barriers remains a persistent challenge in research, education, and clinical practice. In many settings, responsibility is often implicitly placed on the service user to adapt linguistically, rather than on the healthcare systems to provide structurally embedded solutions [5, 8]. This dynamic may reflect broader structural inequities, where responsibility for overcoming communication barriers is shifted onto already disadvantaged populations. As a result, providers frequently rely on ad hoc strategies, such as informal interpreting by family members or bilingual staff, or the use of gestures, when qualified interpreters are unavailable [9–11]. While pragmatic, these approaches may compromise accuracy, confidentiality, and quality of care [10, 12]. Although qualified interpreters are considered the preferred standard, their availability is often limited by logistical, legal, financial and organisational constraints [13–15].

In this context, translation technologies, including fixed-phrase, machine translation tools and all applications (apps) or devices that function as a direct language mediator between service user and service provider, have emerged as accessible and readily available alternatives to support communication in multilingual encounters [16, 17]. These tools vary substantially in their underlying architecture, accuracy, regulatory status, and suitability for clinical use, ranging from general-purpose machine translation systems to specialised medical applications. This heterogeneity has implications for how their use and acceptance should be interpreted. These tools can facilitate rapid, on-demand communication and are increasingly integrated into everyday practice. From a health equity perspective, such tools may offer opportunities to expand access to at least basic communication in settings where formal language support is limited. However, existing research indicates substantial variability in their performance and usability. Translation accuracy may differ across languages and clinical contexts, with particular challenges observed in the translation of complex or service user-specific information [18, 19]. More broadly, the evidence base highlights inconsistencies in evaluation approaches and a lack of standardised frameworks [17].

Complementing this evidence, qualitative research suggests that while translation technologies are valued for their immediacy and accessibility, they have important limitations in supporting the relational and contextual dimensions of care. Studies indicate that such tools may struggle to convey empathy, cultural nuance, and contextual meaning, and may disrupt conversational flow in sensitive interactions [20–23]. Concerns regarding data protection and the use of commercially available applications further influence acceptance, particularly in contexts with strict privacy regulations [20, 22, 23]. In addition, successful implementation appears to depend not only on individual acceptance but also on organisational factors, including the availability of appropriate infrastructure, training and clear institutional guidance [16, 17, 24]. Existing evidence suggests that translation technologies are increasingly used in practice, but their role in shaping equitable communication and care for linguistically diverse service users remains insufficiently understood [17, 23].

Despite the growing use of translation technologies in healthcare, there remains limited quantitative evidence on how healthcare providers use these tools in routine practice, how they evaluate their usefulness, and which contextual and organisational factors shape their adoption. In this sense, their increasing use may reflect ongoing structural gaps in the provision of formal language support, rather than a systematically integrated solution. Such evidence is essential to inform the safe, appropriate, and equitable integration of translation technologies into diverse healthcare systems, and to assess their potential role in addressing, or potentially reproducing, existing inequities in healthcare access and quality.

The Technology Acceptance Model (TAM) is a widely used framework for examining individuals’ acceptance and intended use of technology [25, 26]. It identifies perceived usefulness (PU) and perceived ease of use (PEOU) as central beliefs related to attitude toward use (ATT) and behavioural intention (BI). TAM was considered relevant to the present study because its core constructs provide a parsimonious framework for examining healthcare providers’ evaluations of translation technologies and their intention to use them in practice.

### Study aims and objectives

Against this background, and in light of ongoing structural gaps in the provision of language support, the present study aims to investigate healthcare providers’ experiences, practices, and attitudes regarding the use of translation technologies in healthcare and psychosocial settings. The specific objectives are to:

1. Describe the frequency and context in which providers encounter language barriers and the strategies they use to overcome them.
2. Assess the acceptance of translation technologies using the TAM constructs PEOU, PU, ATT, and BI.
3. Describe contextual, ethical, and organisational factors relating to translation technology use, including perceived accuracy, privacy concerns, organisational encouragement, and conditional access to translation technologies.
4. Explore variation in communication practices across professional groups and country contexts, with a primary focus on the German sample and exploratory comparisons across countries.
5. Identify factors associated with BI to use translation technologies, including TAM constructs and selected contextual, organisational, demographic, and professional variables.

## Methods

### Study design and setting

The study employed a cross-sectional online survey as part of the international research project “Multilingualism in providing quality mental healthcare to migrants – needs, resources and practices” (MiM2M). A cross-sectional design was chosen to capture a snapshot of healthcare providers’ communication practices, experiences with translation technologies, and attitudes towards their use at a single point in time.

Data were collected between 30 September and 31 December 2025 using the LimeSurvey platform (University of Hamburg license). The study included healthcare and psychosocial service providers working in Germany, the Netherlands, Romania, and South Africa.

The study was originally designed to enable cross-country comparisons across all participating sites. However, due to differences in recruitment outcomes and resulting sample size imbalances, the analytical strategy was adapted before inferential modelling. Inferential analyses were restricted to the German sample to ensure stable model estimation, while Romanian data were retained for exploratory and descriptive comparisons to provide contextual insights.

### Participants and recruitment

Eligible participants were healthcare and psychosocial service providers aged 18 years or older who, based on self-assessment, regularly encountered service users with limited proficiency in the dominant language(s) of the respective healthcare system. The eligibility criteria were stated in the recruitment materials and study information. No predefined frequency threshold or separate screening item was used to verify eligibility.

Participants were recruited using convenience and snowball sampling. Invitations were distributed via institutional mailing lists, professional associations, social media, and printed materials in healthcare settings. Recruitment was conducted in each participating country by members of the respective research teams using similar strategies adapted to local contexts. Participants were encouraged to share the survey with eligible colleagues. Because these open recruitment and snowball-sampling procedures did not permit determination of how many individuals received or encountered an invitation, a conventional survey response rate could not be calculated.

Participation was voluntary and anonymous. After receiving study information and providing informed consent, participants completed the questionnaire in their preferred language. Completion time was approximately five to ten minutes. No personal identifying data were collected, and no incentives were provided.

### Sample size determination

An a priori power analysis indicated that approximately 169 participants per country were required to detect medium-sized between-country differences (Cohen’s f = 0.25) with 80% power at a significance level of α = 0.05. This resulted in a target sample size of 676 participants across four countries.

As this target was not achieved across all countries, the study was not sufficiently powered for the originally intended cross-country inferential comparisons, and such analyses were therefore treated as exploratory.

The final inferential analysis focused on multivariable regression modelling within the German sample (*n* = 258). The planned regression models included up to eight variables, resulting in more than 30 observations per predictor, which exceeds commonly recommended thresholds (10–20 observations per variable) for stable model estimation.

### Survey instrument

The questionnaire was developed in English through an iterative, team-based process informed by a prior scoping review [17], preliminary qualitative interview findings [23], TAM literature [25, 26], and related research [27]. Items assessing TAM constructs were developed based on existing TAM literature and adapted to the study context.

The interdisciplinary research team included psychologists, psychotherapists, medical doctors, an epidemiologist, linguists, and public health researchers. This range of expertise informed the iterative development and internal piloting of the questionnaire, particularly the review of item relevance and clarity. Following internal piloting, the final version was translated into German, Romanian, and Dutch by native-speaking researchers. A forward-translation approach was used, and translated versions were reviewed within the research team to support conceptual equivalence across languages. Back-translation was not conducted, and cross-language measurement invariance was not formally assessed. Comparisons of scale scores between language and country samples were treated as exploratory and descriptive. The translated versions were pilot-tested with healthcare providers in each country, resulting in minor refinements. Pilot testing focused on assessing clarity, comprehensibility, and relevance of items; however, formal cognitive debriefing procedures were not conducted.

The final questionnaire comprised 43 items across 10 thematic sections, including language barriers, communication strategies, use of translation technologies, acceptance constructs, contextual and ethical considerations, institutional conditions, and demographic characteristics.

### Measures and variables

The primary outcome variable was BI to use translation technologies, operationalised as the mean score of multiple Likert-scale items adapted from the TAM. Behavioural intention refers to the extent to which healthcare providers indicate a willingness or plan to use translation technologies in their future clinical practice. All TAM constructs were measured using 5-point Likert scales (1 = strongly disagree to 5 = strongly agree).

Key independent variables included the core TAM constructs: PU, PEOU, and ATT, reflecting the cognitive and affective orientation of translation technologies. The inclusion of these constructs was theory-driven, based on the TAM, which has been applied to explain technology adoption. While alternative frameworks incorporate additional contextual and organisational factors, TAM was selected due to its conceptual parsimony and suitability for capturing individual-level acceptance within the constraints of a brief survey instrument.

Additional contextual variables included perceived accuracy and privacy concerns. Language-related variables captured exposure to language barriers, linguistic context of care, and multilingual background.

Participants also reported their use of communication strategies and specific translation technologies, including modality (text, voice, or both) and functional scope (e.g., single terms, full sentences, entire conversations).

Institutional variables included organisational encouragement, conditional access to translation technologies, training availability, organisational guidance, access to devices, and internet reliability. Demographic and professional characteristics (e.g., country, profession, work experience, gender) were included as potential covariates. Selection of these variables was guided by a combination of theoretical considerations (TAM and prior literature) and their conceptual relevance as potential determinants or confounders of behavioural intention. A detailed overview of the analysis plan, including variable operationalisation and mapping of survey items, is provided in Supplementary File 1.

### Ethical considerations

The study was conducted in accordance with the ethical standards of the Declaration of Helsinki. Ethical approval was obtained from the ethics committee of the University Medical Center Hamburg-Eppendorf, Germany (Reference number: LPEK-0935). Ethical oversight for all participating sites was coordinated through the lead institution. All participants provided informed consent prior to participation. Participants were informed about the purpose of the study, the voluntary nature of participation, and their right to withdraw at any time without consequences. No identifying personal data were collected, and survey responses were stored securely on university servers in compliance with the European Union General Data Protection Regulation (EU-GDPR).

The reporting of this study followed the Strengthening the Reporting of Observational Studies in Epidemiology (STROBE) guidelines for cross-sectional studies, with the checklist included as Supplementary File 3 [28].

### Data analysis

The quantitative analyses were conducted in R (version 4.5.2) [29]. An analysis plan was prepared a priori by AK to ensure consistency and transparency across data preparation, scale construction, and analyses, and was refined iteratively as data exploration and analysis progressed.

Due to differences in sample size across countries, analyses were restricted to participants from Germany and Romania, with inferential modelling conducted in the German sample.

### Data preparation and missing data

Data were screened for completeness, internal consistency, and outliers. Duplicate entries and logically inconsistent responses were removed prior to analysis. All pilot data were excluded before the official launch of the survey.

Patterns of missingness were examined descriptively. Survey completion status was determined using the completion status recorded by the survey platform. A substantial proportion of participants discontinued the questionnaire before completing key sections, particularly those containing the items required to construct the primary outcome, BI, and the core predictor variables. Consequently, these participants could not be included in the main analyses.

To assess potential attrition bias, completers and non-completers were compared using variables available before survey discontinuation. Because participants discontinued the questionnaire at different stages, the number of available observations varied across comparisons. Categorical variables were compared using chi-square tests, with Cramér’s V reported as an effect-size measure. Continuous or ordinal variables were compared using Wilcoxon rank-sum tests. These comparisons were necessarily restricted to information provided before discontinuation.

For multi-item scales, composite scores were calculated using a ≥ 75% item-completion rule. Scale scores were set to missing if the completion threshold was not met. For regression analyses, a complete-case approach was adopted, including only participants with available data on all variables included in the respective models.

### Composite scale construction and psychometric evaluation

For each TAM construct (PU, PEOU, ATT, BI), item responses were aggregated into composite scale scores. Internal consistency of multi-item subscales PU and PEOU was assessed using Cronbach’s alpha. For the two-item subscales ATT and BI, inter-item correlations and Spearman-Brown reliability coefficients were calculated, because Cronbach’s alpha is less informative for two-item measures. Values ≥ 0.70 were considered indicative of acceptable internal consistency. Estimates below this threshold were retained but interpreted cautiously. Composite scores were calculated as mean values when at least 75% of items were completed. No confirmatory factor analysis was conducted.

### Descriptive statistics

Descriptive analyses were used to summarise sample characteristics, exposure to language barriers, the linguistic context of care, use of communication strategies, access to translation technologies, and institutional and infrastructural conditions across the analytic sample and professional groups. For continuous variables, normality was assessed via visual inspection of histograms and Q-Q plots; normally distributed data are presented as means with standard deviations (SD), while skewed data are reported as medians with interquartile ranges (IQR).

Preliminary analyses indicated contextual heterogeneity between the two recruitment sites, including differences in exposure to language barriers and evaluation of technical infrastructure. Therefore, inferential modelling was restricted to the German sample, while Romanian data were used for exploratory and descriptive comparisons. Country-specific TAM scores were summarised descriptively. Because measurement invariance across language versions was not assessed, these values were not interpreted as evidence of equivalent construct-level differences between countries.

### Group comparisons and preparatory analyses

Exploratory group comparisons were conducted across professional groups and gender. Other country-specific patterns, including communication strategies and technology use, were examined descriptively. Differences across professional groups and gender were assessed using independent t-tests or ANOVA, with effect sizes reported as Cohen’s *d* or η^2^. Given the exploratory nature of these subgroup analyses and the number of comparisons conducted, no formal correction for multiple testing was applied. Reported p-values should therefore be interpreted with caution.

For the German sample, preparatory analyses examined missingness patterns, internal consistency of scales (Cronbach’s α), and bivariate associations (Pearson’s *r)*. These analyses were used to explore relationships between variables and to inform model specification based on both empirical associations and their conceptual relevance within the TAM-informed framework.

### Regression analyses

Factors associated with BI were examined using ordinary least squares (OLS) regression. BI was calculated as the mean of two five-point Likert items and had nine possible values. OLS was retained to facilitate interpretation of standardised coefficients and comparability with previous TAM-based research. Models were estimated in two steps: a core model containing primary TAM constructs (PEOU, PU, ATT), and an adjusted model incorporating demographic, organisational, and contextual variables.

Given the cross-sectional design and analytical approach, the TAM constructs were included as parallel variables in the models. Accordingly, the regression coefficients were interpreted as controlled associations rather than as total effects corresponding to the causal pathways specified in the original TAM framework. In particular, the estimated association of PEOU with BI may be attenuated when controlling for PU and ATT because of the theorised relationships between these constructs. The models were therefore not intended to test mediation or the directional pathways proposed in the TAM.

Given the bounded and discrete distribution of BI, model assumptions were assessed using residual-versus-fitted and Q-Q plots, the Breusch-Pagan test for heteroscedasticity, the Shapiro-Wilk test of residual normality, and Cook’s distance for potentially influential observations. Because the diagnostics indicated heteroscedasticity, heteroscedasticity-consistent HC3 standard errors, confidence intervals, and p-values were used for the main inferential reporting.

As a sensitivity analysis, the adjusted model was additionally estimated using proportional-odds ordinal logistic regression, treating the nine observed BI values as ordered categories. Continuous predictors were standardised so that their corresponding odds ratios represented the association with a one-standard-deviation increase in the respective predictor. This analysis was used to assess whether the substantive pattern of findings was robust to modelling BI as an ordered rather than continuous outcome.

Covariate selection for the primary adjusted model was informed by theoretical considerations, prior literature, and the intended focus on organisational and contextual factors beyond the core TAM constructs. Age was retained as a general demographic covariate. The primary adjusted model was not intended to provide exhaustive adjustment for all demographic and professional characteristics. Gender, work experience, mental-healthcare setting, and professional group were not included simultaneously in this model to preserve parsimony, given the conceptual overlap between age and work experience and between professional group and healthcare setting, as well as the additional parameters required for the multi-category professional-group variable.

Two additional sensitivity analyses examined the implications of these modelling decisions. First, reported exposure to language barriers was added to the primary adjusted model. Second, an expanded model included all variables from the primary adjusted model together with exposure to language barriers, gender, work experience, mental-healthcare setting, and professional group. Continuous predictors were standardised, and HC3 heteroscedasticity-consistent confidence intervals and p-values were used. Multicollinearity was assessed using variance inflation factors (VIFs); all values in the primary adjusted model were below 2.3. Statistical significance was set at *p* < 0.05. However, given the exploratory nature of the expanded sensitivity model and subgroup analyses, the corresponding p-values should be interpreted with caution.

## Results

### Participant flow and attrition analysis

Of the 777 participants who initiated the survey, 461 completed it (59.3%), while 316 discontinued before completion (40.7%). Among the 461 completed questionnaires, 258 were from participants working in Germany, 185 from Romania, 13 from South Africa, and five from the Netherlands. Because of the small sample sizes from South Africa and the Netherlands, these 18 completed questionnaires were excluded from further analyses, resulting in a final German-Romanian analytic sample of 443 participants.

Comparisons between completers and non-completers were based on information available before survey discontinuation and therefore involved varying sample sizes across variables (Supplementary File 2, Table S9). There was no evidence of differences in country distribution (*p* = 0.694), mental-healthcare setting (*p* = 0.730), or reported exposure to language barriers (*p* = 0.112). Completers reported slightly more work experience than non-completers (median: 13 vs. 10 years; *p* = 0.029). The groups also differed in professional composition (*p* < 0.001, Cramér’s V = 0.226) and workplace setting (*p* < 0.001, Cramér’s V = 0.239). Because only subsets of non-completers had reached the corresponding questions, these comparisons should be interpreted cautiously.

### Sample characteristics

The final analytic sample comprised 443 participants, of whom 258 were working in Germany and 185 in Romania (see Table 1). Participants had a mean age of 42.3 years (*SD* = 12.0, range 19–76) and were predominantly female (72.2%). Overall, 43.3% (*n* = 192) of participants reported that they worked primarily in mental healthcare, with comparable proportions observed in Germany and Romania. Participants reported an average of 15.5 years of work experience in healthcare (*M* = 15.5, *SD* = 11.5). Mean work experience was descriptively higher among respondents in Romania (*M* = 17.5, *SD* = 11.9) than among those in Germany (*M* = 14.0, *SD* = 11.1).

The professional composition of the sample reflected a wide range of roles within the healthcare system, categorised into six primary functional groups. Psychosocial health professionals (39.5%, *n* = 175) represented the largest group and included psychotherapists, psychologists, and social workers. Medical doctors (24.6%, *n* = 109) comprised general practitioners and various medical specialists.

The category of medical-technical and other health professions (16.5%, *n* = 73) exhibited a high degree of geographical and professional concentration. In the German sample, this group represented a substantial portion of participants (27.9%, *n* = 72) and was composed almost exclusively of paramedics and emergency medical technicians, whereas it was virtually absent in the Romanian sample (0.5%, *n* = 1). This distribution indicates a strong overlap between professional group, country, and workplace setting (e.g., emergency medical services), which limits the interpretability of profession-based comparisons across countries.

Nursing staff (9.9%, *n* = 44) included registered, paediatric, and other nursing specialisations. Therapeutic professions (4.7%, *n* = 21) referred to providers of physical and functional therapies, such as physiotherapists and occupational therapists. Administrative staff (3.8%, *n* = 17) included medical assistants and hospital administrative personnel.

Participants reported working across a wide range of healthcare and related service settings (see Table 1). While hospitals (29.1%, *n* = 75) and emergency medical services (26.4%, *n* = 68) were more frequently reported in Germany, private practice and practitioner-led settings were more common in Romania (36.2%, *n* = 67).

The German and Romanian samples differed in several characteristics relevant to the interpretation of country-specific patterns. Participants in the German sample were younger on average than those in the Romanian sample (*M*: 39.5 vs. 46.2 years), and the proportion of female participants was lower in Germany (64.7%) than in Romania (82.7%). Professional composition also differed, with medical doctors representing 14.7% of the German sample and 38.4% of the Romanian sample. Conversely, the medical-technical group consisted almost entirely of German participants. All subsequent comparisons between the country samples are unadjusted and exploratory. Observed patterns may therefore reflect differences in sample composition as well as differences between the healthcare contexts.

**Table 1:** Sample characteristics of healthcare providers by country

Characteristic	Overall	Germany	Romania
*N*	443	258	185
**Age** (years), *M* (*SD*); min-max	42.3 (12.0); 19–76	39.5 (11.8); 19–76	46.2 (11.1); 23–73
**Gender**			
Female	320 (72.2%)	167 (64.7%)	153 (82.7%)
Male	112 (25.3%)	85 (32.9%)	27 (14.6%)
Non-binary	4 (0.9%)	3 (1.2%)	1 (0.5%)
Prefer not to answer	7 (1.6%)	3 (1.2%)	4 (2.2%)
**Primary work in mental healthcare or psychosocial care**			
No	251 (56.7%)	141 (54.7%)	110 (59.5%)
Yes	192 (43.3%)	117 (45.3%)	75 (40.5%)
**Work experience in healthcare** (years), *M (SD*); min-max	15.5 (11.5); 0–50	14.0 (11.1); 1–50	17.5 (11.9); 0–46
**Profession group**			
Psychosocial health professions	175 (39.5%)	100 (38.8%)	75 (40.5%)
Medical doctors	109 (24.6%)	38 (14.7%)	71 (38.4%)
Nursing staff	44 (9.9%)	31 (12.0%)	13 (7.0%)
Medical-technical and other health professions	73 (16.5%)	72 (27.9%)	1 (0.5%)
Administrative staff	17 (3.8%)	10 (3.9%)	7 (3.8%)
Therapeutic professions	21 (4.7%)	4 (1.6%)	17 (9.2%)
Other	4 (0.9%)	3 (1.2%)	1 (0.5%)
**Workplace setting**			
Ambulatory nursing services	3 (0.7%)	2 (0.8%)	1 (0.5%)
Community health centre	25 (5.6%)	16 (6.2%)	9 (4.9%)
Education/research setting	18 (4.1%)	3 (1.2%)	15 (8.1%)
Emergency medical services	68 (15.3%)	68 (26.4%)	0
Hospital	120 (27.1%)	75 (29.1%)	45 (24.3%)
Mental health facility	36 (8.1%)	24 (9.3%)	12 (6.5%)
Nursing home/long-term care facility	8 (1.8%)	5 (1.9%)	3 (1.6%)
Outpatient clinic	25 (5.6%)	15 (5.8%)	10 (5.4%)
Private practice/practitioner’s practice	107 (24.2%)	40 (15.5%)	67 (36.2%)
Public health department	17 (3.8%)	9 (3.5%)	8 (4.3%)
Rehabilitation centre	7 (1.6%)	1 (0.4%)	6 (3.2%)
Other	9 (2.0%)	0	9 (4.9%)

Continuous variables are presented as means, standard deviations, and ranges; categorical variables are presented as *n* (%). Percentages were calculated within each country. Due to rounding, percentages may not sum to 100%

### Linguistic context of care

Participants reported encountering a wide range of languages in their daily work, reflecting diverse multilingual care contexts. Across the analytic sample, the most frequently reported languages, excluding the primary languages of the respective healthcare systems, included English (63.4%, *n* = 281), Arabic (26.4%, *n* = 117), and Turkish (21.7%, *n* = 96). These patterns highlight differences in the linguistic composition of service user populations between the two country contexts.

In Germany, participants most commonly reported encounters with Arabic (*n* = 116), Turkish (*n* = 94), and Russian (*n* = 81), whereas in Romania, encounters with English (*n* = 128), Hungarian (*n* = 50), and French (*n* = 23) predominated. While the primary languages of care (German and Romanian) were frequently mentioned by participants due to the open wording of the survey item, they reflect variation in the linguistic composition of service user populations across contexts. In the German sample, frequently encountered languages are often less likely to be shared by providers and may therefore pose more substantial communication challenges. In contrast, the high frequency of English in the Romanian sample may, in some cases, reflect the use of a shared lingua franca rather than a complete language barrier, as providers may have at least partial proficiency in English. These differences suggest that the nature and intensity of language barriers may vary across settings and may not be directly comparable based on frequency alone.

Overall, these language profiles, based on the three most frequently encountered languages, provide a descriptive overview of the linguistic environment. They serve as a helpful background for understanding the diversity of communication demands across settings, rather than constituting formal outcome measures or a comprehensive inventory of all languages spoken in these healthcare contexts.

### Exposure to language barriers

Participants reported substantial variability in their exposure to language barriers in daily practice. Across the analytic sample, respondents estimated that a median of 10% (IQR = 24) of their work-related interactions during the previous three months were affected by language barriers.

A large difference in reported exposure was observed between the country samples: participants working in Germany reported a substantially higher typical exposure (median = 20%, IQR = 22.5) than those working in Romania (median = 1%, IQR = 5) (see Table 2). These findings suggest that differences between the two contexts are not only quantitative but may also reflect differences in how language barriers are perceived and reported in everyday practice. In the German sample, language barriers are likely to arise more frequently in encounters with languages not shared by providers. In contrast, in the Romanian context, the high frequency of languages such as English or Hungarian may indicate situations in which providers have at least partial proficiency, and where cross-linguistic communication may not consistently be perceived or reported as a barrier. It is important to note that exposure was assessed through self-reported estimates and reflects participants’ subjective experiences. As such, what constitutes a language barrier may vary between individuals and contexts. Similarly, the reporting of encountered languages, based on open-ended responses, may be influenced by salience, such that widely used lingua franca (e.g., English) may not always be explicitly considered when recalling language use. These factors should be considered when interpreting differences in exposure between country samples.

**Table 2:** Self-reported exposure to language barriers in healthcare encounters by country and professional group

		*N*	M (SD)	Median (Q1, Q3)	Min	Max
**Country** ^α^						
Overall		443	18.0 (24.1)	10 (1, 25)	0	100
Germany		258	25.4 (25.9)	20 (7.5, 30)	0	100
Romania		185	7.8 (16.9)	1 (0, 5)	0	100
**Professional group** ^**α**^						
Medical-technical		73	21.7 (16.5)	20 (10, 30)	0	100
Nursing staff		44	19.8 (23.8)	15 (2, 20)	0	100
Psychosocial health professions		175	19.6 (26.3)	5.5 (1, 30)	0	100
Administrative staff		17	16.6 (25.6)	1 (0, 25)	0	80
Medical doctors		109	14.6 (24.9)	3 (1, 20)	0	100
Therapeutic professions		21	6.2 (12.3)	1 (0, 5)	0	50
Other		4	30 (52.0)	0 (0, 45)	0	90

^α^Missing values for exposure: Overall (*n* = 4); Germany (*n* = 3), Romania (*n* = 1); Medical-technical *(n* = 1), Nursing staff (*n* = 1), Psychosocial health (*n* = 1), Other (*n* = 1). The “Other” category (*n* = 4) is too small for meaningful statistical summary; values should be interpreted with caution

Exposure also varied across professional groups, with the highest typical rates reported by medical-technical professions (median = 20%, IQR = 20) and nursing staff (median = 15%, IQR = 18). In contrast, medical doctors (median = 3%, IQR = 19) and therapeutic professions (median = 1%, IQR = 5) reported lower typical exposure. Psychosocial health professionals exhibited the greatest within-group variability. Despite a relatively low median exposure (5.5%), the large interquartile range (IQR = 29) indicates that their experience is highly dependent on specific workplace contexts, with exposure rates for the top quarter of this group reaching 30% or higher.

### Use of strategies to overcome language barriers

Participants utilised a diverse range of strategies to bridge communication gaps in the three months preceding the survey, with technological tools emerging as the primary resource in daily practice. Across the total sample, translation applications were the most prevalent strategy, reported by 65.0% of providers (*n* = 288) (see Table 3). This was followed by communication in a shared language (54.2%, *n* = 240) and informal interpreting by the service user’s adult family members or friends (49.0%, *n* = 217).

These findings reflect overall reported use and should be interpreted in the context of differences in exposure to language barriers between countries. In particular, the higher exposure reported in the German sample may partly explain the greater use of communication strategies.

Disparities in recent practice were observed between countries. Providers in Germany reported a markedly higher use of translation apps (74.0%) and shared-language communication (62.0%) than those in Romania (52.4% and 43.2%, respectively). The use of qualified interpreters showed the sharpest divide: 30.6% of German participants utilised those services in the past three months, compared with 4.3% of the Romanian participants. Furthermore, the involvement of underage family members as informal interpreters was reported more often in the German sample (38.0% vs. 3.2% in Romania).

**Table 3:** Use of communication strategies to overcome language barriers among healthcare providers by country

Strategy	Overall (*n*, %)	Germany (*n*, %)	Romania (*n*, %)
Translation apps	288 (65.0%)	191 (74.0%)	97 (52.4%)
Use of a shared language	240 (54.2%)	160 (62.0%)	80 (43.2%)
Service user’s family/friends serving as interpreters	217 (49.0%)	146 (56.6%)	71 (38.4%)
Bilingual staff/colleagues help out as interpreters	166 (37.5%)	123 (47.7%)	43 (23.2%)
Service user’s *underage* family/friends serving as interpreters	104 (23.5%)	98 (38.0%)	6 (3.2%)
Qualified interpreters	87 (19.6%)	79 (30.6%)	8 (4.3%)
Use of visual aids	83 (18.7%)	61 (23.6%)	22 (12.0%)
Multilingual material	78 (17.6%)	57 (22.1%)	21 (11.4%)

Multiple responses were possible for strategy; percentages therefore do not sum to 100%. Percentages refer to the proportion of participants selecting each option. For country-specific columns, proportions were calculated using country-specific denominators

Strategy use also varied across professional groups (see Supplementary File 2, Table S1). Nursing staff (75.0%) and medical-technical health professions (87.7%) reported the highest recent uptake of translation applications. These findings should be interpreted with caution, as professional group membership is closely associated with country and workplace setting, particularly for medical-technical staff (e.g., paramedics), which were almost exclusively represented in the German sample. Accordingly, findings for this group primarily reflect the German emergency care context rather than a cross-national professional category. In contrast, psychosocial health professionals reported lower rates of app use (53.1%) and informal family interpreting (29.1%), relying more heavily on shared-language communication (56.0%). Across all professional roles, formal solutions like qualified interpreters (19.6%) and multilingual materials (17.6%) remained the least common strategies utilised during the reporting period.

### Use of translation technologies

Experience with translation technologies was nearly universal across the sample, with Google Translate emerging as the dominant tool, utilised by 82.8% of all participants (*n* = 367) (see Table 4). In contrast, DeepL showed a distinct regional preference, with experience reported by 37.2% of participants in Germany compared to only 10.8% in Romania. While online dictionaries were utilised by 14.9% of providers (*n* = 66), adoption of specialised medical translation technologies (e.g., Aidminutes) and other applications remained marginal, with each used by fewer than 3% of participants. Some of these differences may reflect contextual factors such as regional availability and market penetration, rather than differences in provider attitudes. In addition, it is important to note that the category of “translation technologies” encompasses a heterogeneous set of tools, including general-purpose machine translation applications, domain-specific tools, and emerging large language model-based systems, which differ in functionality, intended use, and underlying technology.

Tool experience also varied by professional role, with medical-technical professions (95.9%) and nursing staff (93.2%) reporting the highest lifetime adoption of Google Translate (see Supplementary File 2, Table S2). Psychosocial health professionals reported comparatively lower, though still majority, usage of the tool (73.7%). The use of large language model (LLM)-based tools, such as ChatGPT, was mentioned by only 4.1% of participants (*n* = 18), suggesting these emerging technologies are not yet a primary resource in clinical workflows. A small minority of providers, with a higher proportion in Romania than Germany (10.8% vs. 5.8%), reported no experience with translation technologies in their professional work.

**Table 4:** Use of translation technologies among healthcare providers by country

Technological tool	Total (*n*, %)	Germany (*n*, %)	Romania (*n*, %)
Aidminutes	11 (2.5%)	11 (4.3%)	0
DeepL	116 (26.2%)	96 (37.2%)	20 (10.8%)
Google Translate	367 (82.8%)	222 (86.0%)	145 (78.4%)
Microsoft Translator	8 (1.8%)	6 (2.3%)	2 (1.1%)
Online dictionary	66 (14.9%)	46 (17.8%)	20 (10.8%)
TalkToMe	4 (0.9%)	2 (0.8%)	2 (1.1%)
iTranslate Converse	6 (1.4%)	3 (1.2%)	3 (1.6%)
Universal Doctor Translator	1 (0.2%)	0	1 (0.5%)
LLM (any)	18 (4.1%)	9 (3.5%)	9 (4.9%)
Vasco	5 (1.1%)	5 (1.9%)	0
Apple translate	4 (0.9%)	4 (1.6%)	0
Other tools (name unknown)	11 (2.5%)	9 (3.5%)	2 (1.1%)
No experience with any translation tool	35 (7.9%)	15 (5.8%)	20 (10.8%)

Multiple responses were possible for technological tool use; percentages therefore do not sum to 100%. Percentages refer to the proportion of participants selecting each option. For country-specific columns, proportions were calculated using country-specific denominators

### Modality of use

Providers utilised translation technologies through different input and output modalities. Overall, a majority of tool-using participants (60.7%, *n* = 216) reported using both written (text) and oral (voice/speech) functions depending on the situation. Approximately 35.1% (*n* = 125) restricted their use to text functions only, while a small minority (4.2%, *n* = 15) used voice functions exclusively.

Modality distributions differed between the two country samples. In Germany, a higher proportion of providers used both functions (68.5%, *n* = 152) compared to Romania, where 47.8% (*n* = 64) used both modalities and 50.0% (*n* = 67) relied solely on written text (see Table 5, Panel A).

**Table 5:** Modality and scope of translation technology use by country

	Total, *N* (%)	Germany, *n* (%)	Romania *n* (%)
**Panel A. Modality among tool users**	*N* = 356	*n* = 222	*n* = 134
Both functions	216 (60.7%)	152 (68.5%)	64 (47.8%)
Text (written) only	125 (35.1%)	58 (26.1%)	67 (50.0%)
Voice/speech (oral) only	15 (4.2%)	12 (5.4%)	3 (2.2%)
**Panel B. Functional scope in the full analytic sample**	*N* = 443	*n* = 258	*n* = 185
Full sentences	271 (61.2%)	184 (71.3%)	87 (47.0%)
Specialised single terms	186 (42.0%)	114 (44.2%)	72 (38.9%)
Entire conversations	118 (26.6%)	99 (38.4%)	19 (10.3%)

Panel A percentages were calculated among participants who actively used translation technologies and provided a modality preference. Panel B percentages were calculated using the full analytic sample and the corresponding country-specific samples. Multiple responses were possible for functional scope; percentages in Panel B therefore do not sum to 100%

Breakdowns by professional group showed that medical-technical professions had the highest rate of multi-modal use at 81.7% (*n* = 58). Medical doctors (63.2%, *n* = 60) and nursing staff (63.9%, *n* = 23) also showed a strong preference for using both functions. Conversely, psychosocial health professionals were the most likely to rely on a single modality, with 51.2% (*n* = 65) using text functions only (see Supplementary File 2, Table S3).

### Functional scope

Functional scope patterns varied between the two country samples. The translation of full sentences was the most prevalent practice in both Germany (71.3%, *n* = 184) and Romania (47.0%, *n* = 87). The translation of specialised single terms was reported by 44.2% (*n* = 114) of German and 38.9% (*n* = 72) of Romanian providers. Conducting entire conversations via translation technologies was more common among German participants (38.4%, *n* = 99) than Romanian participants (10.3%, *n* = 19) (see Table 5, Panel B).

Analysis by profession indicated that medical-technical staff utilised these tools most extensively across all categories, including full sentences (86.3%, *n* = 63), entire conversations (54.8%, *n* = 40), and specialised terms (49.3%, *n* = 36). Nursing staff (68.2%, *n* = 30) and medical doctors (63.3%, *n* = 69) also frequently translated full sentences. Among psychosocial health professionals, the primary use case was full sentences (51.4%, *n* = 90), while conducting entire conversations was reported by 11.4% (*n* = 20) of this group.

### Institutional and infrastructural conditions

Formal institutional support for the use of translation technologies was limited across the analytic sample (see Table 6). Only 3.6% (*n* = 16) of participants reported that their workplace provided explicit guidelines for the use of translation technologies in service user-provider communication, while 22.8% reported that they did not know whether such guidelines existed. Similarly, formal training was almost entirely absent, with only 2.7% (*n* = 12) of respondents having received instruction on tool use, despite a strong expressed desire for such training (*M* = 3.7, *SD* = 1.1).

Perceived organisational support and infrastructure varied between countries. Participants reported low levels of active workplace encouragement to use translation apps (*M* = 2.8, *SD* = 1.1). However, agreement was high regarding the potential for increased usage if workplaces provided access to advanced translation technologies (*M* = 3.7, *SD* = 1.0). Regarding infrastructure, 77.8% (*n* = 335) of participants described their internet connection as stable. Romanian providers frequently reported “very stable” connections compared to their German counterparts (54.1% vs. 23.6%, respectively).

Access to digital hardware was mixed; while 42.2% (*n* = 187) of participants had regular access to workplace-provided devices, a slightly larger proportion (43.6%, *n* = 193) relied on personal devices for clinical translation. Several obstacles to implementation were identified (see Table 6). Concerns about accuracy (58.2%) and data privacy (40.9%) were the most frequently cited barriers, followed by a lack of access to reliable tools (29.6%) and insufficient training (26.9%). Notably, 25.4% of Romanian participants perceived no obstacle to tools use, compared to only 3.1% of participants in Germany.

**Table 6:** Institutional and infrastructural conditions for the use of translation technologies by country

	Total	Germany	Romania
**Guidelines**			
Yes	3.6%	5.0%	1.6%
No	73.6%	65.9%	84.3%
I don’t know	22.8%	29.1%	14.1%
**Training received**			
Yes	2.7%	3.5%	1.6%
No	97.3%	96.5%	98.4%
**Internet stability**			
Very stable	161 (36.3%)	61 (23.6%)	100 (54.1%)
Mostly stable	184 (41.5%)	125 (48.4%)	59 (31.9%)
Occasionally unstable	60 (13.5%)	44 (17.1%)	16 (8.6%)
Frequently unstable	26 (5.9%)	21 (8.1%)	5 (2.7%)
No internet access	12 (2.7%)	7 (2.7%)	5 (2.7%)
**Device access**			
No, I use my personal device	193 (43.6%)	114 (44.2%)	79 (42.7%)
Yes, regularly	187 (42.2%)	102 (39.5%)	85 (45.9%)
Sometimes	43 (9.7%)	29 (11.2%)	14 (7.6%)
No access to digital devices at work	20 (4.5%)	13 (5.0%)	7 (3.8%)
**Perceived obstacles**			
Concerns about accuracy	258/443; 58.2%	180/258; 69.8%	78/185; 42.2%
Data privacy issues	181/443; 40.9%	136/258; 52.7%	45/185; 24.3%
Lack of access to reliable tools	131/443; 29.6%	97/258; 37.6%	34/185; 18.4%
Lack of training on how to use translation technologies	119/443; 26.9%	88/258; 34.1%	31/185; 16.8%
Limited language options	92/443; 20.8%	53/258; 20.5%	39/185; 21.1%
Poor internet connection	92/443; 20.8%	75/258; 29.1%	17/185; 9.2%
There are no obstacles	55/443; 12.4%	8/258; 3.1%	47/185; 25.4%
**Desire for training** (*M*,* SD*)	3.7 (1.1)	3.6 (1.1)	3.8 (1.0)
**Organisational encouragement to use translation apps** (*M*,* SD*)	2.8 (1.1)	2.5 (1.0)	3.2 (1.0)
**Organisational provision of translation technologies** (*M*,* SD*)	3.7 (1.0)	3.7 (1.0)	3.6 (1.0)

Multiple responses were possible for perceived obstacles; percentages therefore do not sum to 100%. Percentages refer to the proportion of participants selecting each option. For country-specific columns, proportions were calculated using country-specific denominators. Mean values for training desire, organisational encouragement, and organisational provision refer to 5-point Likert scales (1 = strongly disagree to 5 = strongly agree)

### Acceptance of translation technologies

Overall, participants reported moderately positive acceptance of translation technologies across all four TAM constructs, with mean scores generally trending toward the “agree” end of the 5-point Likert scale (see Table 7). Mean scores were highest for BI (*M* = 3.8, *SD* = 0.9) and ATT (*M* = 3.8, *SD* = 0.8), followed closely by PU (*M* = 3.7, *SD* = 0.7). In contrast, PEOU yielded the lowest evaluation (*M* = 3.3, *SD* = 0.9), indicating more neutral perceptions and greater variability regarding the usability of these tools.

Descriptively, mean TAM scores were higher in the Romanian sample than in the German sample across all four constructs. For example, the mean PEOU score was 3.7 (*SD* = 0.8) in the Romanian sample and 3.0 (*SD* = 0.8) among the German providers, while the corresponding BI means were 3.9 (*SD* = 0.8) and 3.7 (*SD* = 0.9), respectively. These country-specific values are presented solely to characterise the two samples. Because cross-language measurement invariance was not assessed, they should not be interpreted as evidence of equivalent construct-level differences between countries.

**Table 7:** Descriptive summary of TAM constructs by country sample

country	Overall	Germany	Romania
N	443	258	185
Perceived usefulness *M (SD)*	3.7 (0.7)	3.5 (0.7)	3.9 (0.7)
Perceived ease of use *M (SD)*	3.3 (0.9)	3.0 (0.8)	3.7 (0.8)
Attitude toward use *M (SD)*	3.8 (0.8)	3.8 (0.9)	3.9 (0.8)
Behavioural intentionI *M (SD)*	3.8 (0.9)	3.7 (0.9)	3.9 (0.8)

Values are presented descriptively as means and standard deviations. SD, standard deviation. Cross-language measurement invariance was not assessed; country-specific scores should therefore not be interpreted as directly comparable estimates of underlying TAM constructs

Within the sample, acceptance appeared to vary by professional role for most constructs. Exploratory group comparisons showed differences in PU (*F*(6,436) = 3.97, *p* = 0.001, η^2^ = 0.05), ATT (*F*(6,436) = 2.50, *p* = 0.022, η^2^ = 0.03), and BI (*F*(6,436) = 2.76, *p* = 0.012, η^2^ = 0.04). In contrast, PEOU showed less evidence of variation by professional group (*F*(6,436) = 1.63, *p* = 0.139, η^2^ = 0.02). Effect sizes were small (η^2^ = 0.02–0.05). Specifically, in the German sample, nursing staff (*M* = 3.8, *SD* = 0.6) and medical-technical staff (*M* = 4.0, *SD* = 0.4) reported the highest levels of intention, whereas psychosocial health professionals reported more neutral scores (*M* = 3.4, *SD* = 0.9).

Analysis of gender groups indicated a pattern of higher technology acceptance among female participants. Exploratory group comparisons showed differences across all core TAM dimensions: female providers reported higher PU (*p* = 0.003), more positive ATT (*p* = 0.001), and a stronger BI (*p* = 0.006) than male providers. Female participants also evaluated PEOU more favourably (*p* = 0.014).

### Preparatory analyses and bivariate associations

Prior to descriptive and inferential analyses, the internal consistency of the TAM constructs was assessed. Internal consistency was acceptable for the multi-item constructs (PU: α = 0.82; PEOU: α = 0.85). The two-item BI scale also demonstrated acceptable reliability (inter-item *r* = 0.74; Spearman-Brown = 0.85). In contrast, the two-item ATT scale showed a moderate inter-item correlation (*r* = 0.45) and a Spearman-Brown coefficient of 0.62, which was below the predefined threshold of 0.70. The corresponding ATT coefficients were 0.61 in the German sample and 0.65 in the Romanian sample (Supplementary File 2, Table S7).

Within the German sample, the core TAM constructs were positively intercorrelated, supporting the internal coherence of the model in this dataset. PU showed the strongest bivariate association with BI, followed by ATT and PEOU. Bivariate analyses further indicated that organisational and contextual variables were more weakly and inconsistently associated with BI and the other TAM constructs. All bivariate associations, including the full correlation matrix of TAM constructs, organisational variables, contextual factors, and BI, are provided in Supplementary File 2 (see Table S4).

### Multivariable regression

#### Sample and modelling approach

Multivariable regression analyses were conducted exclusively within the German sample (*n* = 257) to examine factors associated with BI to use translation technologies. The analytical strategy utilised two ordinary least squares (OLS) models: Model 1 assessed core TAM constructs, while Model 2 adjusted for organisational and demographic factors. Results and model fit indices are presented in Table 8.

#### Model 1: TAM core

In model 1, all three TAM constructs were positively associated with BI. PU showed the largest standardised coefficient among the TAM constructs (β = 0.44, 95% CI: 0.29–0.60, *p* < 0.001), while ATT (β = 0.23, 95% CI: 0.12–0.34, *p* < 0.001) and PEOU (β = 0.18, 95% CI: 0.04–0.32, *p* = 0.010) showed smaller positive associations. The ATT coefficient should be interpreted cautiously because the reliability of the two-item ATT scale was below the predefined threshold. The model accounted for approximately 55% of the variance in behavioural intention (*R*^*2*^ = 0.554).

#### Model 2: Adjusted model

The second, adjusted model incorporated organisational conditions, contextual concerns, and age to assess the stability of the associations observed in Model 1. The magnitude and direction of the associations for the TAM constructs remained largely unchanged, with PU continuing to show the largest standardised coefficient with BI (see Table 8).

None of the additional organisational or concern-related variables showed statistically significant controlled associations with BI after adjustment. Conditional access to translation technologies was positively but not statistically significantly associated with BI (β = 0.08, 95% CI: -0.02-0.18, *p* = 0.116). Similarly, organisational encouragement, accuracy concerns, and privacy concerns were not statistically associated with BI in the adjusted model. Age showed a small negative association that did not reach statistical significance (β = -0.07, 95% CI: -0.15-0.002, *p* = 0.058).

Overall, the inclusion of contextual factors resulted in only a small increase in explained variance beyond the core TAM constructs, as reflected in the explained variance and model fit indices. While the adjusted model accounted for a slightly higher proportion of variance in behavioural intention (*R*^*2*^ = 0.569; *R*^*2*^_*adj*_ = 0.555), AIC and BIC values indicated no improvement in model fit compared to the core model. This pattern does not demonstrate that organisational conditions and contextual concerns are unimportant, as their adjusted coefficients may partly reflect variance shared with the TAM constructs. Multicollinearity diagnostics indicated no evidence of problematic multicollinearity (all VIF < 2.3; see Supplementary File 2, Table S8).

**Table 8:** OLS linear regression analyses with HC3-robust inference and model fit

Variables	Model 1 β (95% CI)	*p*-value	Model 2 adj. β (95% CI)	*p*-value
Perceived ease of use	0.18 (0.04–0.32)	0.010	0.18 (0.04–0.32)	0.011
Perceived usefulness	0.44 (0.29–0.60)	< 0.001	0.42 (0.26–0.59)	< 0.001
Attitude toward use	0.23 (0.12–0.34)	< 0.001	0.22 (0.10–0.34)	< 0.001
Encouragement			-0.02 (-0.12-0.08)	0.715
Conditional access			0.08 (-0.02-0.18)	0.116
Accuracy concern			0.03 (-0.16-0.22)	0.764
Privacy Concern			-0.04 (-0.21-0.13)	0.640
Age			-0.07 (-0.15-0.002)	0.058
**Model fit**				
*R²*	0.554		0.569	
Adjusted *R²*	0.549		0.555	
AIC	530.576		532.232	
BIC	548.322		567.723	

Both models were estimated in the common complete-case sample (*n* = 257). Coefficients are standardised β estimates from OLS regression. Confidence intervals and p-values are based on HC3 heteroscedasticity-consistent standard errors. OLS, ordinary least squares; AIC, Akaike information criterion; BIC, Bayesian information criterion. Model fit is reported as *R²*, adjusted *R²*, AIC, and BIC. Statistical significance was set at *p* < 0.05

#### Sensitivity analyses

In the German sample, 13.2% of participants had the maximum BI score, indicating some clustering at the upper end of the scale. Residual diagnostics for the adjusted OLS model indicated heteroscedasticity (Breusch-Pagan *X*^*2*^ = 24.20, *p* = 0.002) and a modest departure from residual normality (Shapiro-Wilk *W* = 0.984, *p* = 0.005). The residual Q-Q plot showed that deviations were concentrated primarily in the tails. Twenty observations exceeded the screening threshold of 4/n for Cook’s distance; however, the maximum Cook’s distance was 0.061 and no observation exceeded 1, providing no indication that the results were driven by a single highly influential case. Residual-versus-fitted and Q-Q plots are presented in Supplementary File 2, Figure S1-S3.

Given the evidence of heteroscedasticity, HC3-robust confidence intervals and p-values were used for inferential interpretation of the OLS models. The substantive pattern was unchanged: PEOU, PU, and ATT remained positively associated with BI, whereas the organisational and contextual variables were not independently associated with BI in the adjusted model.

The ordinal logistic sensitivity analysis produced a consistent pattern of results (Supplementary File 2, Table S5). A one-standard-deviation increase in PEOU was associated with higher odds of reporting a higher BI category (OR = 1.61, 95% CI: 1.16–2.25, *p* = 0.005). Corresponding associations were observed for PU (OR = 3.89, 95% CI: 2.63–5.76, *p* < 0.001) and ATT (OR = 2.00, 95% CI: 1.48–2.71, *p* < 0.001). The ATT estimate should be interpreted cautiously given the sub-threshold reliability of the ATT scale. The organisational and contextual variables were not statistically significant in the ordinal model. Thus, modelling BI as an ordered outcome did not materially change the substantive conclusions.

Adding exposure to language barriers to the adjusted model did not materially change the TAM coefficients. Exposure itself was not associated with BI (β = -0.002, 95% CI: -0.10-0.09, *p* = 0.972). PEOU (β = 0.18, 95% CI: 0.04–0.33, *p* = 0.011), PU (β = 0.42, 95% CI: 0.25–0.59, *p* < 0.001), and ATT (β = 0.22, 95% CI: 0.11–0.34, *p* < 0.001) remained positively associated with BI.

Results were also broadly consistent in the expanded sensitivity model including exposure, gender, work experience, mental-healthcare setting, and professional group (*n* = 254; Supplementary File 2, Table S11). PU (β = 0.44, 95% CI: 0.26–0.61, *p* < 0.001) and ATT (β = 0.20, 95% CI: 0.07–0.32, *p* = 0.002) remained positively associated with BI, while the PEOU coefficient was smaller and its confidence interval approached zero (β = 0.15, 95% CI: 0.0004-0.29, *p* = 0.049). Exposure to language barriers, gender, work experience, and mental healthcare setting were not statistically significantly associated with BI in this model. Among the professional groups, medical doctors showed a higher BI score than the reference group of psychosocial health professionals (β = 0.46, 95% CI: 0.10–0.82, *p* = 0.012); the remaining professional-group estimates were not statistically significant. Given the exploratory nature of this expanded model and the small numbers in some professional groups, these estimates should be interpreted cautiously. The expanded model accounted for 60.4% of the variance in BI (*R*^*2*^=0.604; adjusted *R*^*2*^=0.572).

## Discussion

### Summary of main findings

This cross-sectional study examined healthcare providers’ use and acceptance of translation technologies in multilingual healthcare settings. The findings indicate that translation technologies are widely used in everyday practice, despite limited formal organisational support. In the German regression sample, behavioural intention was most strongly associated with perceived usefulness. This may reflect the perceived value of translation technologies for addressing immediate communication needs, although this interpretation was not directly tested. At the same time, variation in use across country samples and professional groups suggests that adoption may be shaped by contextual factors, including differences in exposure to language barriers and communicative demands. Behavioural intention was more closely associated with the TAM constructs than with the organisational variables included in the adjusted model, while technology use occurred in contexts with limited formal support. Finally, the findings highlight important limitations of translation technologies in relational forms of communication and raise concerns regarding their implications for equitable care.

### Pragmatic adoption: translation technologies as coping strategies

The widespread use of translation technologies observed in this study suggests that their adoption can be understood as a pragmatic response to persistent language barriers in everyday clinical practice. Rather than being systematically implemented, these tools appear to function as ad hoc solutions that enable healthcare providers to maintain communication when formal resources, such as interpreting services, are unavailable.

This reliance on ad hoc solutions is not limited to digital tools. A notable proportion of participants also reported the use of minors as interpreters in recent clinical encounters, particularly in the German sample. The use of minors as interpreters is widely discouraged due to concerns regarding accuracy, role conflict, and potential psychological burden on the child [4, 30]. While the present study does not provide detailed contextual information on these situations, this finding further illustrates how healthcare providers may resort to readily available but suboptimal communication strategies when formal resources are limited. Taken together, these patterns suggest that both technological and non-technological coping mechanisms emerge in response to gaps in structured language support. This aligns with previous research describing the use of informal strategies to manage language barriers in resource-constrained settings [10, 13].

Importantly, this pattern may reflect a trade-off between immediacy and optimal communication conditions. Providers appear to use translation technologies not because they are considered ideal, but because they are readily available and allow basic communication to proceed. This interpretation is consistent with studies in emergency and primary care contexts, where translation technologies are used to support more task-oriented communication despite recognised limitations [31–33].

In this sense, translation technologies function less as formally adopted innovations and more as embedded coping mechanisms within systems where structural solutions to language barriers remain insufficient. Adoption in this sense is shaped by necessity rather than preference, highlighting the role of translation technologies as situated responses to gaps in service provision rather than fully integrated components of care.

### Technology acceptance and the role of perceived usefulness

Beyond this situational necessity, the findings provide insight into factors associated with providers’ willingness to use translation technologies. Consistent with TAM-informed expectations, PU showed the largest controlled association with behavioural intention, while PEOU and ATT showed smaller positive associations. However, the ATT finding should be interpreted cautiously because this construct was assessed using a two-item scale with sub-threshold reliability. These findings should also be interpreted in light of the heterogeneous nature of the technologies included in this study, which differ in functionality and intended use. The comparatively large PU coefficient is consistent with the possibility that perceived support for clinical tasks is particularly relevant to providers’ stated intention to use these technologies. However, the cross-sectional associations do not demonstrate that perceived usefulness drives adoption or that providers prioritise usefulness over usability. This pattern aligns with TAM-based research identifying perceived usefulness as an important correlate of technology-adoption intentions in professional contexts where tools are evaluated based on their contribution to task performance [25–27].

The comparatively weaker role of perceived ease of use may reflect the widespread familiarity and accessibility of commonly used translation applications, as observed in this study and previous research [17]. In contexts where tools are readily available and require minimal training, this familiarity may be one possible explanation for the smaller PEOU coefficient relative to PU, although this was not directly examined.

The organisational and ethical variables did not show statistically significant controlled associations with behavioural intention in the adjusted model. This should not be understood as evidence that organisational conditions are unimportant. Previous research has emphasised the relevance of infrastructure, institutional guidance, and governance for the implementation of translation technologies in healthcare [17, 21, 24]. In the present study, conditional access was associated with BI at the bivariate level (*r* = 0.25), but this association was attenuated after the TAM constructs were included. One possible explanation is that organisational conditions are partly reflected in providers’ assessments of whether technologies are useful and feasible in practice. Another is that the generally limited availability of formal support, guidelines, and training reduced the variation needed to identify separate associations. Similarly, accuracy and privacy concerns may influence which technologies providers use and in which situations, rather than their general intention to use translation technologies. The present data cannot distinguish between these explanations or establish indirect effects. Longitudinal research would be needed to examine how organisational conditions, individual evaluations, and behavioural intention develop over time.

### Translation technologies as informal digital infrastructure

While the previous sections describe individual-level patterns of technology use and acceptance, the findings also reveal a structural gap in how their use is supported and governed within healthcare systems. Despite widespread use in clinical practice, formal organisational frameworks, such as guidelines, approved tools, or structured training, were largely absent. In addition, many providers relied on personal devices rather than institutionally provided technologies.

This constellation of findings suggests that translation technologies may currently operate as a form of informal digital infrastructure within multilingual healthcare settings. Rather than being systematically integrated into healthcare delivery, these tools are primarily used as ad hoc solutions driven by individual initiative. This pattern resonates with the concept of “shadow IT”, which describes the use of unapproved or non-sanctioned technologies by employees in the absence of adequate institutional provision. In healthcare settings, related phenomena have been described as “workarounds” or informal technology use, whereby healthcare providers circumvent or temporarily fix workflow constraints in order to maintain care delivery under challenging conditions [34]. Such practices are typically driven by practical necessity but raise concerns regarding standardisation, data security, and accountability. Similar observations have been reported in previous research, where translation technologies have been described as workarounds in the absence of institutional support or clear guidance [16, 17, 23]. The present findings extend this perspective by showing that, within the study sample, widespread use occurred alongside limited formal organisational support. This pattern suggests that translation technologies may already function as informal digital infrastructure in some healthcare settings.

The growing availability of artificial intelligence-based translation technologies further amplifies this gap between practice and governance. Policy analyses and recent interdisciplinary research indicate that such tools are being adopted in healthcare contexts more quickly than evidence-based regulatory frameworks are being developed to guide their safe, equitable, and appropriate use [24, 35]. Concerns have been raised in particular regarding communicative reliability, accountability, and the ethical use of these technologies in high-stakes settings. In this context, individual healthcare providers effectively assume responsibility for decisions regarding tool selection, use, and risk management, despite limited institutional support [24]. Addressing this mismatch may require healthcare systems to move beyond ad hoc adoption toward more structured implementation strategies that include validated tools, clear guidance, and integration with professional interpreting services.

### Contextual heterogeneity: exposure, profession, and the dynamics of tool use

The findings further indicate that the use and acceptance of translation technologies are not uniform but vary across clinical and organisational contexts. Descriptive differences observed between country samples and professional groups suggest that patterns of adoption may be associated with variations in exposure to language barriers, communicative demands, and working environments.

Mean TAM scores were consistently higher in the Romanian sample across all TAM constructs. While this may suggest a more favourable orientation toward translation technologies, several alternative explanations should be considered. First, cross-cultural differences in Likert-scale response styles may have contributed to systematically higher scores, as response tendencies such as reduced central tendency or greater use of extreme categories have been documented across contexts. Second, given the comparatively low reported exposure to translation technologies in the Romanian sample, participation may reflect a degree of self-selection, whereby individuals with a more positive disposition toward such tools were more likely to respond. Third, lower levels of direct experience may also limit exposure to potential challenges or failures of these technologies in clinical practice, potentially resulting in more favourable evaluations. These factors should be considered when interpreting cross-country differences in acceptance [36, 37].

In the sensitivity analyses, exposure to language barriers was not associated with behavioural intention, and including it did not change the TAM coefficients. However, the analysis concerned intended future use rather than actual use. In addition, the exposure measure captured how frequently providers encountered language barriers, but not the complexity or communicative demands of these encounters. The result therefore does not rule out that exposure influences the use of translation technologies in practice.

Professional roles also appear to be related to how translation technologies are used. Providers engaged in time-sensitive, task-oriented communication, such as emergency or acute care, may perceive these tools as particularly useful for exchanging concrete information where clarity and speed are prioritised [31, 33]. In contrast, providers working in more dialogue-based settings may adopt them more cautiously. This may be related to the greater communicative complexity of their interactions and the need for nuanced understanding [7, 23].

However, these observations should be interpreted with caution, as professional roles were unevenly distributed across country contexts. In particular, the medical-technical group was almost exclusively composed of German paramedics and emergency medical staff, indicating a strong overlap between profession, country, and workplace setting. As a result, apparent profession-related differences may partly reflect contextual factors, such as exposure to language barriers, organisational conditions, and communicative demands, rather than independent effects of professional role.

These patterns suggest that perceived usefulness may vary with context and task demands rather than representing a fixed attribute of translation technologies. This aligns with broader evidence indicating that the perceived value of such tools varies depending on clinical context and communicative function [22, 23]. While these interpretations remain exploratory, they underscore the importance of considering contextual variation when examining the adoption and use of translation technologies in healthcare.

### Relational dimensions and the limits of technological mediation

While the previous sections highlight the patterns associated with the use of translation technologies, the findings also point to important boundaries of their applicability. In particular, translation technologies appear to be less suited for forms of communication that rely on emotional nuance, relational trust, and contextual understanding.

The more cautious adoption patterns observed among psychosocial professionals may be consistent with concern about their suitability for relational communication. In clinical contexts such as mental healthcare, communication extends beyond the exchange of factual information and involves emotionally and culturally embedded interaction that shapes care processes and outcomes. In such settings, interaction itself becomes a core component of care rather than merely a vehicle for information transfer [7, 38].

Existing research supports this distinction between functional and relational communication. While translation technologies may facilitate basic information exchange, their effectiveness appears to be largely confined to task-oriented communication, with more limited capacity to support nuanced, context-dependent interaction, or sustained dialogue. Even when general comprehension is achieved, important aspects of meaning such as tone or intention may be simplified or lost [22, 23, 32].

These findings suggest that translation technologies are selectively useful depending on the communicative task. While they may support initial or routine exchanges, more complex interactions may continue to require human mediation.

### Equity implications: opportunities and risks of translation technologies

The findings of this study identify several conditions that may be relevant to the equitable implementation of translation technologies, although the study did not directly assess inequalities in communication quality or healthcare outcomes. Rather than functioning as neutral tools, their use appears to be embedded within uneven structural and individual conditions, raising concerns about how benefits and limitations may be distributed across clinical encounters. In the present study, 43.6% of participants reported using personal devices, while only 3.6% reported the availability of institutional guidelines and 2.7% had received training. In addition, 58.2% reported concerns about translation accuracy and 40.9% about data privacy. These findings indicate that translation technologies are frequently used under conditions of limited standardised organisational support.

The reliance on personal devices and individual judgement could contribute to variation in how translation technologies are selected and used across clinical encounters. However, the present study did not assess providers’ digital literacy, device quality, or the resulting quality of communication. It therefore cannot determine whether variation between providers resulted in unequal communication experiences for service users. Nevertheless, when the selection and use of translation technologies depend largely on individual providers rather than shared organisational standards, variability in communication support remains a plausible equity concern.

These concerns are further reinforced by evidence that the performance of machine translation varies across languages and types of clinical content, with lower accuracy often observed for less-resourced or linguistically complex languages [16, 17, 39]. In the present study, participants reported working with a wide range of languages, including Arabic and Persian, which are often underrepresented in training datasets or present structural challenges for machine translation systems [18, 19, 40]. While this study did not assess translation accuracy directly, the combination of diverse linguistic demand and variable tool performance suggests that communication quality may differ across linguistic groups.

The largely informal and unregulated nature of translation technology use observed in this study may further contribute to these patterns. When providers rely on personal devices and individual judgement in the absence of organisational guidance, the selection and use of tools may vary considerably across settings. Policy analyses have highlighted that artificial intelligence-based translation technologies are being adopted in healthcare settings more rapidly than governance frameworks are being developed to guide their safe and equitable implementation [24]. In this context, ensuring equitable multilingual care may require not only access to translation technologies but also institutional strategies that support appropriate tool selection, training, and integration with professional interpreting services. If translation technologies are used without systematic evaluation or validation across linguistic contexts, there is a risk that some service user groups may receive less accurate or less reliable communication support than others [17].

Translation technologies may play a valuable role in expanding communication possibilities in multilingual healthcare, particularly in settings where alternative resources are limited. However, their contribution to equitable care will depend on how they are implemented, evaluated, and governed within healthcare systems. Without such safeguards, their increasing use may contribute to new forms of variability in communication quality, with implications for fairness and safety in healthcare delivery.

### Strengths and limitations

This study has several strengths. It addresses an emerging and under-researched area by examining the use and acceptance of translation technologies in multilingual healthcare settings. While previous research has primarily focused on translation accuracy or specific technological tools, this study provides insight into how such technologies are currently used in everyday clinical practice and how healthcare providers perceive their usefulness. It combines theory-informed analysis with empirical data by applying constructs from the TAM to examine factors associated with behavioural intention to use translation technologies. This approach allows the findings to be interpreted within a broader theoretical framework of technology adoption in healthcare.

The study includes a diverse sample of healthcare providers across different professional roles and country contexts. This diversity enables the identification of variation in use and acceptance across settings, offering a more nuanced understanding of how translation technologies are embedded in everyday clinical practice.

The study captures a snapshot of real-world practices, including the use of translation technologies in the absence of formal organisational support. By reflecting everyday clinical realities, the findings provide relevant insights into how communication challenges are currently managed in practice, and highlight gaps between individual use and institutional frameworks.

Several limitations should be considered when interpreting the findings. First, while the study was initially designed to enable cross-country analyses, this aim could not be realised as intended. The study design was adapted, resulting in a primary focus on the German sample and a more exploratory inclusion of data from Romania. Data from other country contexts, including the Netherlands and South Africa, were not included due to small sample sizes. As a result, the regression analyses reflect a single-country context rather than an international comparison, and cross-country differences should be interpreted with caution. Observed variations may reflect contextual factors, but may also be influenced by differences in sample size and recruitment dynamics.

Second, the cross-sectional design does not allow for causal inferences regarding the relationships observed between technology acceptance constructs and behavioural intention. The identified associations should therefore be interpreted as indicative rather than explanatory of underlying mechanisms. In addition, the use of adapted and brief measures limits the psychometric evidence available for the TAM constructs. No confirmatory factor analysis was conducted, and the two-item ATT scale demonstrated sub-threshold reliability overall (Spearman-Brown coefficient: 0.62) and in the German inferential sample (Spearman-Brown = 0.61). Measurement error may reduce the precision of estimates and complicate interpretation of the ATT coefficient. Although ATT was retained because it was a pre-specified component of the TAM-informed model, its association with behavioural intention should be interpreted cautiously. The relatively high proportion of variance explained by the regression models should likewise be interpreted with caution. Although the multicollinearity diagnostics did not indicate problematic collinearity, they cannot exclude overlap in item content or common-method variance among the self-reported TAM constructs. The parallel-predictor models were not designed to test mediation or establish the directional relationships proposed in the TAM.

Third, the study relies on self-reported data, which may be subject to recall bias or social desirability bias. Participants’ reported use of translation technologies and perceptions of their usefulness may not fully reflect actual behaviour in clinical interactions. In addition, subgroup comparisons were conducted on an exploratory basis and were not adjusted for multiple testing, increasing the risk of type I error; these findings should therefore be interpreted with caution.

Fourth, the questionnaire underwent forward translation and team review, but no back-translation or formal assessment of measurement invariance across language versions was conducted. It therefore remains uncertain whether the TAM scales measured the same constructs in an equivalent manner across the German and Romanian versions. Observed differences in country-specific mean scores may reflect measurement artefacts, differences in response patterns, or sample composition rather than underlying differences in technology acceptance. Accordingly, all cross-country TAM comparisons are exploratory and descriptive.

Fifth, the study did not assess the objective accuracy or quality of the translation technologies, limiting the ability to draw conclusions about their effectiveness in practice. A related limitation concerns the operationalisation of “translation technologies” as a single category in the inferential analyses. This grouping included a heterogeneous set of tools, ranging from general-purpose machine translation (e.g., Google Translate), to domain-specific tools (e.g., Aidminutes), as well as large language model-based systems. These tools differ substantially in terms of functionality, accuracy, data processing, and intended use. As a result, the reported levels of acceptance may reflect aggregated perceptions across distinct technologies rather than evaluations of specific tool types. Future research would benefit from more fine-grained analyses that distinguish between classes of translation technologies.

Sixth, the sample composition and recruitment approach may limit the generalisability of the findings. Although the inclusion of multiple countries and professional groups is a strength, the distribution of participants across contexts was uneven, and convenience and snowball sampling were used. Because the number of potentially eligible individuals reached through the open recruitment channels was unknown, a conventional response rate could not be calculated. Eligibility was based on participants’ self-assessment and was not verified through a separate screening item or predefined frequency threshold. Participants may therefore have interpreted “regularly” encountering language barriers differently. Moreover, voluntary participation may have resulted in an overrepresentation of individuals with an interest in multilingual communication or digital tools. Consequently, the findings are most directly applicable to the German healthcare context and should be interpreted with caution when considering broader generalisation.

Seventh, a substantial proportion of individuals who initiated the questionnaire did not complete it. Comparisons based on the available data indicated differences between completers and non-completers in professional composition, workplace setting, and work experience, suggesting that attrition may not have been entirely random. However, these comparisons were limited because non-completers discontinued the survey at different stages and demographic variables located near the end of the questionnaire were generally unavailable for this group. Consequently, residual attrition bias cannot be excluded, and the analytic sample may not fully represent all individuals who initiated the survey.

### Implications for practice

The findings suggest that translation technologies have become a pragmatic component of everyday clinical practice in multilingual healthcare settings. Given their widespread use, healthcare providers may benefit from guidance on how to use these tools appropriately and safely. In particular, training initiatives could focus on helping providers identify situations in which translation technologies may be suitable and where alternative strategies, including qualified interpreting, are more appropriate.

In addition, raising awareness of the limitations of translation technologies, particularly in relation to nuanced communication and accuracy, may support more informed decision-making in clinical encounters. Rather than positioning these tools as substitutes for qualified interpreting, they can be understood as complementary resources within a broader repertoire of formal communication strategies.

However, for such practices to be applied consistently and safely, they need to be supported by organisational structures and formalised through policy and institutional guidance.

### Implications for policy and healthcare organisations

At the organisational level, the findings highlight a gap between widespread individual use of translation technologies and the limited availability of formal guidance or institutional support. Healthcare institutions may therefore need to move beyond ad hoc use toward more structured approaches to implementation. This could include the identification and provision of validated tools, the development of clear usage guidelines, and the integration of translation technologies into existing communication support systems and workflows.

Given the potential risks related to data protection, accuracy, and variability across languages, governance frameworks will be essential to ensure safe and equitable use. Recent regulatory developments, such as the European Union’s AI Act (2024), which introduces a risk-based classification for AI systems, highlight the increasing relevance of governance in this area [41]. Translation technologies used in clinical contexts may fall within these considerations, particularly where they influence communication relevant to care. Policies should address not only which tools are appropriate for clinical use, but also how they should be used in different contexts. In addition, ensuring continued access to professional interpreting services remains critical, particularly for complex or sensitive interactions that cannot be adequately supported by technological solutions.

### Implications for research

The findings point to several priorities for future research. First, further studies are needed to evaluate the performance of translation technologies across different languages and clinical contexts, particularly in relation to less-resourced languages that are frequently encountered in practice. Such research would help to better understand the conditions under which these tools can support safe and effective communication.

Second, longitudinal and observational studies could provide deeper insight into how translation technologies are used in real clinical interactions and how their use influences communication processes and outcomes over time. This would complement the self-reported data used in the present study and allow for a more comprehensive assessment of their role in healthcare delivery.

Third, future research should explore how translation technologies can be integrated into healthcare systems in a way that supports both effectiveness and equity. This includes examining how organisational policies, training, and infrastructure shape the use of these tools, as well as how they interact with existing resources such as qualified interpreting services.

Finally, comparing research across countries and healthcare systems would be valuable to better understand how contextual factors influence the adoption and use of translation technologies. Differences in organisational structures, resource availability, and patterns of language diversity may provide important insights into how these tools are integrated into clinical practice and under which conditions they are most effective.

## Conclusions

This study highlights the growing role of translation technologies in multilingual healthcare as pragmatic tools used to address immediate communication needs in the absence of formal support. Translation technologies were commonly used, while perceived usefulness showed the largest controlled association with behavioural intention in the German sample. Use varied across contexts, and widespread adoption occurred alongside limited formal guidance and training. Although translation technologies may expand communication possibilities, this study dud not assess their accuracy or effects on care. Their safe and equitable use therefore requires attention to the contexts in which they are used, clear institutional guidance, and continued access to professional interpreting for complex or sensitive interaction. Further research should examine how these approaches affect communication quality and the experiences of service users facing language barriers.

## Supplementary Information

Below is the link to the electronic supplementary material.

Supplementary Material 1: Analysis plan and variable operationalisation. Detailed analysis plan and overview of the operationalisation and mapping of variables used in the study.

Supplementary Material 2: Additional quantitative analyses. Supplementary tables and figures presenting additional descriptive, diagnostic, and sensitivity analyses.

Supplementary Material 3: STROBE checklist. Completed STROBE checklist for cross-sectional studies.

## Data Availability

The datasets used and/or analysed during the current study are available from the corresponding author on reasonable request.

## Abbreviations

AIC: Akaike information criterion
ATT: Attitude toward use
BI: Behavioural intention
BIC: Bayesian information criterion
CI: Confidence interval
EU-GDPR: European Union General Data Protection Regulation
HC3: Heteroscedasticity-consistent standard error estimator
IQR: Interquartile range
LLM: Large language model
OLS: Ordinary least squares
OR: Odds ratio
PEOU: Perceived ease of use
PU: Perceived usefulness
SD: Standard deviation
STROBE: Strengthening the Reporting of Observational Studies in Epidemiology
TAM: Technology Acceptance Model
VIF: Variance inflation factor
