# Supplementary material for: Healthcare providers’ use and acceptance of translation technologies in multilingual healthcare settings: a cross-sectional study
Source: Int J Equity Health. 2026 Oct 21;25:252. doi: 10.1186/s12939-026-03085-6 (PMC13688893; doi:10.1186/s12939-026-03085-6)
Supplement: Supplementary file 1 [file 12939_2026_3085_MOESM1_ESM.pdf]

Supplementary file 1. Healthcare providers' use and acceptance of translation technologies in multilingual healthcare: A cross-sectional study

A: Analysis plan

1. Data preparation and cleaning

Raw survey data and metadata were imported into R (version 4.5.2). Only completed questionnaires were retained for analysis.

Likert-scale items were recoded to numeric values prior to analysis. A unique respondent identifier was generated, and country indicators were created to distinguish between study sites.

Due to differences in recruitment outcomes and resulting sample sizes, analyses focused on participants from Germany and Romania, with inferential analyses restricted to the German sample (see Section 7).

2. Scale construction and psychometric evaluation

Composite scores were constructed for the four core constructs of the Technology Acceptance Model (TAM):

- Perceived Usefulness (PU)
- Perceived Ease of Use (PEOU)
- Attitude Toward Use (ATT)
- Behavioural Intention (BI)

Scale scores were calculated as mean values when at least 75% of items within a scale were completed; otherwise, scores were set to missing.

Internal consistency was assessed as follows:

- Cronbach's alpha for multi-item scales (PU, PEOU), with $\alpha \geq 0.70$ considered acceptable
- Inter-item correlations and Spearman-Brown coefficients for two-item scales (ATT,BI)

Psychometric properties were evaluated for the combined sample and stratified by country.

All composite scores were treated as continuous variables in subsequent analyses.

3. Conceptual framework

A conceptual framework was developed to represent hypothesised relationships between key variables included in the study, including TAM constructs (PEOU, PU, ATT, BI), organisational factors (e.g., encouragement, access to tools), contextual concerns (e.g., perceived accuracy, privacy concerns), and background characteristics (e.g., age, profession, work experience).

The framework was informed by TAM theory and prior empirical literature and was used to guide variable selection and model specification for the regression analyses. It reflects assumed relationships between variables but was not intended as a formal causal model.

4. Descriptive analyses

Descriptive analyses were conducted to characterise the study sample and contextual variables, including:

- Sociodemographic and professional characteristics (age, gender, profession, work experience)
- Exposure to language barriers
- Languages encountered in clinical practice
- Multilingual background
- Use of communication strategies
- Use and modalities of translation technologies
- Institutional and infrastructural conditions

Descriptive statistics were reported overall and stratified by country and/or professional group where appropriate.

The distribution of continuous variables was assessed using visual inspection of histograms and Q-Q plots. Normally distributed variables are presented as means with standard deviations (SD), while skewed variables are summarised using medians and interquartile ranges (IQR).

5. Group comparisons and bivariate analyses

Group comparisons and bivariate analyses were conducted to explore differences and associations between key variables.

- Independent t-tests were used for comparisons between two groups
- One-way analysis of variance (ANOVA) was used for comparisons across multiple groups
- Pearson correlation coefficients were calculated to assess associations between continuous variables

Binary contextual variables (e.g., perceived accuracy and privacy concerns) were examined using t-tests comparing groups with and without reported outcomes.

Gender differences in behavioural intention and TAM constructs were analysed using t-tests or ANOVA, depending on the number of groups.

Additional exploratory analyses included comparisons of behavioural intention across professional groups.

These analyses were primarily descriptive and exploratory in nature and were used to contextualise observed patterns and inform subsequent modelling.

6. Preparatory analyses and model diagnostics

Preparatory analyses within the German sample examined missing data patterns, scale reliability, and bivariate associations between behavioural intention, the TAM constructs, and selected organisational and contextual variables. These analyses informed the interpretation and specification of the regression models alongside theoretical and conceptual considerations.

Given that behavioural intention was calculated as the mean of two five-point Likert items and therefore had nine possible values, the distribution of the outcome and the assumptions of the adjusted ordinary least squares (OLS) model were examined. Diagnostics included residual-versus-fitted and normal Q-Q plots, the Breusch-Pagan test for heteroscedasticity, the Shapiro-Wilk test of residual normality, and Cook’s distance to identify potentially influential observations. The diagnostic results are presented in Supplementary file 2, Table S10 and Figures S1–S3.

7. Multivariable regression analyses

Multivariable OLS regression was used to examine factors associated with behavioural intention (BI) to use translation technologies within the German sample. BI was treated as a continuous outcome derived from the mean of two five-point Likert items. TAM constructs were entered as parallel predictors; their coefficients therefore represent controlled associations rather than total effects or tests of the directional pathways proposed in the original TAM.

Two primary models were estimated:

- **Model 1 (TAM core model):** BI regressed on perceived ease of use (PEOU), perceived usefulness (PU), and attitude toward use (ATT).
- **Model 2 (adjusted model):** Model 1 extended by organisational encouragement, conditional access to translation technologies, accuracy concerns, privacy concerns, and age.

The adjusted model focused on selected organisational and contextual factors and was not intended to provide exhaustive adjustment for demographic and professional characteristics. Continuous predictors were standardised before modelling. Regression analyses used complete cases for the variables included in each model.

Because model diagnostics indicated heteroscedasticity, HC3 heteroscedasticity-consistent confidence intervals and *p* values were used for the main inferential reporting. Model fit was described using R^2, adjusted R^2, AIC, and BIC. Variance inflation factors were examined for multicollinearity.

8. Sensitivity and robustness analyses

Three supplementary analyses examined the robustness and scope of the adjusted model.

1. Proportional-odds ordinal logistic regression was used to model the nine observed BI values as ordered categories. This analysis assessed whether treating BI as an ordered rather than continuous outcome changed the substantive pattern of associations (Supplementary file 2, Table S5).

2. Self-reported exposure to language barriers was added to the primary adjusted OLS model to examine whether its inclusion changed the estimated associations between the TAM constructs and BI.

3. An expanded OLS model included the variables from the primary adjusted model together with exposure to language barriers, gender, work experience, mental-healthcare setting, and professional group. This model assessed the sensitivity of the findings to broader demographic and professional adjustment. HC3 heteroscedasticity-consistent confidence intervals and *p* values were used for both additional OLS models. Their results are presented in Supplementary file 2, Table S11.

The additional models were interpreted as sensitivity analyses. In particular, estimates for individual professional groups in the expanded model were interpreted cautiously because some groups contained few participants.

9. Statistical software and reporting

All analyses were conducted using R (version 4.5.2). Statistical significance was set at $p < 0.05$.

Reporting followed the Strengthening the Reporting of Observational Studies in Epidemiology (STROBE) guidelines for cross-sectional studies.

10. Deviations from the initial analysis plan

An initial analysis plan was prepared before data analysis and was refined as data exploration and analysis progressed. Before inferential analysis, the focus was restricted to the German sample because recruitment outcomes and sample sizes differed across countries. Romanian data were retained for exploratory descriptive reporting.

During manuscript revision, additional analyses were conducted in response to peer-review comments. These included residual diagnostics for the adjusted OLS model, the use of HC3-robust inference for the reported OLS results, an ordinal logistic sensitivity analysis, and two additional OLS sensitivity models examining exposure to language barriers and broader demographic and professional adjustment. Comparisons between survey completers and non-completers were also added to assess potential attrition bias.

These analyses are identified as additional or sensitivity analyses in the manuscript and Supplementary file 2. They should not be interpreted as components that were specified in the initial analysis plan.

B: Variable operationalisation and mapping

Construct / Variable	Description	Operationalisation	Survey items/ source
Behavioural intention (BI)	Intention to use translation technologies in clinical practice	Mean score (2 items), ≥75% completed	g06q33_sq008, g06q33_sq012
Perceived usefulness (PU)	Perceived benefits of using translation technologies	Mean score (5 items), ≥75% completed	g06q33_sq001–sq004, sq013
Perceived ease of use (PEOU)	Perceived ease of using translation technologies	Mean score (4 items), ≥75% completed	g06q33_sq005–sq007, sq009
Attitude toward use (ATT)	Overall evaluation of using translation technologies	Mean score (2 items), ≥75% completed	g06q33_sq010, g06q33_sq011
Organisational encouragement	Perceived encouragement from organisation to use translation technologies	Single Likert-scale item	g09q40_sq001
Conditional access to tools	Availability of translation tools depending on context or conditions	Single Likert-scale item	g09q40_sq002
Accuracy concerns	Concerns about accuracy of translation technologies	Binary variable (0 = not selected, 1 = selected)	g05q32_sq003
Privacy concerns	Concerns about data protection and privacy	Binary variable (0 = not selected, 1 = selected)	g05q32_sq004

Age	Participant age in years	Continuous variable (derived)	Derived from birth year (g10q44) and survey date
Work experience	Years of professional experience in healthcare	Continuous variable	work_exp_healthcare_yrs
Profession group	Professional role in healthcare	Categorical variable (recoded)	g01q02_recoded_label
Gender	Self-reported gender	Categorical variable	gender_label
Exposure to language barriers	Proportion of work involving language barriers	Continuous (%) variable	g02q21_pct
Languages encountered	Languages encountered in clinical practice	Multiple response variable	g02q22_sq001–sq003
Multilingual background	Number of first languages spoken by participant	Categorical variable	n_first_langs_cat
Use of communication strategies	Strategies used to overcome language barriers	Multiple response variables	g05q26_*
Use of translation technologies	Use of digital translation tools in practice	Multiple response variables + free-text flags	g05q28_*
Institutional conditions	Availability of organisational support structures	Categorical variables	g07q37_label, g07q38_label
Infrastructure	Access to devices and internet in workplace	Categorical variables	g09q40_sq001, g09q40_sq002
