# Supplementary material for: Healthcare providers’ use and acceptance of translation technologies in multilingual healthcare settings: a cross-sectional study
Source: Int J Equity Health. 2026 Oct 21;25:252. doi: 10.1186/s12939-026-03085-6 (PMC13688893; doi:10.1186/s12939-026-03085-6)
Supplement: Supplementary file 2 [file 12939_2026_3085_MOESM2_ESM.pdf]

Supplementary file 2: Healthcare providers' use and acceptance of translation technologies in multilingual healthcare: A cross-sectional study.

Additional quantitative analyses

- Table S1. Use of communication strategies by professional group

| Profession group | Communication strategy | *n* | N total profession group | % |
|---|---|---|---|---|
| Psychosocial health professions | Use of a shared language | 98 | 175 | 56 |
| Psychosocial health professions | Translation apps (e.g. Google Translate) | 93 | 175 | 53.14 |
| Psychosocial health professions | Service user's family/friends serving as interpreters | 51 | 175 | 29.14 |
| Psychosocial health professions | Professional interpreters | 48 | 175 | 27.43 |
| Psychosocial health professions | Use of translated written document | 40 | 175 | 22.86 |
| Psychosocial health professions | Bilingual staff/colleagues help out as interpreters | 37 | 175 | 21.14 |
| Psychosocial health professions | Use of visual aids | 36 | 175 | 20.57 |
| Psychosocial health professions | Service user's underage family/friends serving as interpreters | 17 | 175 | 9.71 |
| Medical doctors | Translation apps (e.g. Google Translate) | 77 | 109 | 70.64 |
| Medical doctors | Service user's family/friends serving as interpreters | 66 | 109 | 60.55 |
| Medical doctors | Use of a shared language | 66 | 109 | 60.55 |
| Medical doctors | Bilingual staff/colleagues help out as interpreters | 54 | 109 | 49.54 |
| Medical doctors | Use of translated written document | 23 | 109 | 21.10 |
| Medical doctors | Service user's underage family/friends serving as interpreters | 18 | 109 | 16.51 |
| Medical doctors | Professional interpreters | 17 | 109 | 15.60 |
| Medical doctors | Use of visual aids | 13 | 109 | 11.93 |
| Nursing staff | Translation apps (e.g. Google Translate) | 33 | 44 | 75.00 |
| Nursing staff | Service user's family/friends serving as interpreters | 32 | 44 | 72.73 |
| Nursing staff | Bilingual staff/colleagues help out as interpreters | 31 | 44 | 70.45 |
| Nursing staff | Use of a shared language | 18 | 44 | 40.91 |
| Nursing staff | Professional interpreters | 13 | 44 | 29.55 |
| Nursing staff | Service user's underage family/friends serving as interpreters | 9 | 44 | 20.45 |
| Nursing staff | Use of visual aids | 9 | 44 | 20.45 |
| Nursing staff | Use of translated written document | 6 | 44 | 13.64 |
| Medical-technical and other health professions | Translation apps (e.g. Google Translate) | 64 | 73 | 87.67 |

| | | | | |
|---|---|---|---|---|
| Medical-technical and other health professions | Service user's underage family/friends serving as interpreters | 59 | 73 | 80.82 |
| Medical-technical and other health professions | Service user's family/friends serving as interpreters | 54 | 73 | 73.97 |
| Medical-technical and other health professions | Use of a shared language | 43 | 73 | 58.90 |
| Medical-technical and other health professions | Bilingual staff/colleagues help out as interpreters | 32 | 73 | 43.84 |
| Medical-technical and other health professions | Use of visual aids | 15 | 73 | 20.55 |
| Medical-technical and other health professions | Use of translated written document | 4 | 73 | 5.48 |
| Medical-technical and other health professions | Professional interpreters | 2 | 73 | 2.74 |
| Therapeutic professions | Translation apps (e.g. Google Translate) | 8 | 21 | 38.10 |
| Therapeutic professions | Service user's family/friends serving as interpreters | 7 | 21 | 33.33 |
| Therapeutic professions | Bilingual staff/colleagues help out as interpreters | 6 | 21 | 28.57 |
| Therapeutic professions | Use of visual aids | 5 | 21 | 23.81 |
| Therapeutic professions | Use of a shared language | 3 | 21 | 14.29 |
| Therapeutic professions | Professional interpreters | 2 | 21 | 9.52 |
| Therapeutic professions | Use of translated written document | 1 | 21 | 4.76 |
| Administrative staff | Translation apps (e.g. Google Translate) | 12 | 17 | 70.59 |
| Administrative staff | Use of a shared language | 11 | 17 | 64.71 |
| Administrative staff | Service user's family/friends serving as interpreters | 6 | 17 | 35.29 |
| Administrative staff | Bilingual staff/colleagues help out as interpreters | 5 | 17 | 29.41 |
| Administrative staff | Professional interpreters | 5 | 17 | 29.41 |
| Administrative staff | Use of visual aids | 4 | 17 | 23.53 |
| Administrative staff | Use of translated written document | 3 | 17 | 17.65 |
| Administrative staff | Service user's underage family/friends serving as interpreters | 1 | 17 | 5.88 |
| Other (free text) | Bilingual staff/colleagues help out as interpreters | 1 | 4 | 25.00 |
| Other (free text) | Service user's family/friends serving as interpreters | 1 | 4 | 25.00 |
| Other (free text) | Translation apps (e.g. Google Translate) | 1 | 4 | 25.00 |
| Other (free text) | Use of a shared language | 1 | 4 | 25.00 |
| Other (free text) | Use of translated written document | 1 | 4 | 25.00 |
| Other (free text) | Use of visual aids | 1 | 4 | 25.00 |

Multiple responses were possible for strategy; percentages therefore do not sum to 100%. Percentages refer to the proportion of participants selecting each option.

- Table S2. Use of translation technologies by professional group

| **Profession group** | **Translation technology** | ***n*** | ***N* total profession** | **%** |
|---|---|---|---|---|

| | | | | |
|---|---|---|---|---|
| Psychosocial health professions | Google Translate | 129 | 175 | 73.71 |
| Psychosocial health professions | DeepL | 47 | 175 | 26.86 |
| Psychosocial health professions | Online dictionary (e.g. Merriam-Webster, LEO) | 30 | 175 | 17.14 |
| Psychosocial health professions | No experience with any translation tool | 22 | 175 | 12.57 |
| Psychosocial health professions | TalkToMe | 2 | 175 | 1.14 |
| Psychosocial health professions | iTranslate Converse | 1 | 175 | 0.57 |
| Medical doctors | Google Translate | 95 | 109 | 87.16 |
| Medical doctors | DeepL | 19 | 109 | 17.43 |
| Medical doctors | Online dictionary (e.g. Merriam-Webster, LEO) | 15 | 109 | 13.76 |
| Medical doctors | No experience with any translation tool | 7 | 109 | 6.42 |
| Medical doctors | Microsoft Translator | 3 | 109 | 2.75 |
| Medical doctors | iTranslate Converse | 3 | 109 | 2.75 |
| Medical doctors | Universal Doctor Translator | 1 | 109 | 0.92 |
| Nursing staff | Google Translate | 41 | 44 | 93.18 |
| Nursing staff | DeepL | 12 | 44 | 27.27 |
| Nursing staff | Online dictionary (e.g. Merriam-Webster, LEO) | 3 | 44 | 6.82 |
| Nursing staff | Microsoft Translator | 2 | 44 | 4.55 |
| Nursing staff | TalkToMe | 2 | 44 | 4.55 |
| Nursing staff | No experience with any translation tool | 1 | 44 | 2.27 |
| Medical-technical and other health professions | Google Translate | 70 | 73 | 95.89 |
| Medical-technical and other health professions | DeepL | 24 | 73 | 32.88 |
| Medical-technical and other health professions | Online dictionary (e.g. Merriam-Webster, LEO) | 11 | 73 | 15.07 |
| Medical-technical and other health professions | Aidminutes | 10 | 73 | 13.70 |
| Medical-technical and other health professions | Microsoft Translator | 3 | 73 | 4.11 |
| Medical-technical and other health professions | iTranslate Converse | 2 | 73 | 2.74 |
| Therapeutic professions | Google Translate | 15 | 21 | 71.43 |
| Therapeutic professions | No experience with any translation tool | 3 | 21 | 14.29 |
| Therapeutic professions | Online dictionary (e.g. Merriam-Webster, LEO) | 3 | 21 | 14.29 |
| Therapeutic professions | DeepL | 2 | 21 | 9.52 |
| Administrative staff | Google Translate | 14 | 17 | 82.35 |
| Administrative staff | DeepL | 9 | 17 | 52.94 |
| Administrative staff | No experience with any translation tool | 2 | 17 | 11.76 |
| Administrative staff | Online dictionary (e.g. Merriam-Webster, LEO) | 1 | 17 | 5.88 |
| Other (free text) | DeepL | 3 | 4 | 75.00 |

| | | | | |
|---|---|---|---|---|
| Other (free text) | Google Translate | 3 | 4 | 75.00 |
| Other (free text) | Online dictionary (e.g. Merriam-Webster, LEO) | 3 | 4 | 75.00 |
| Other (free text) | Aidminutes | 1 | 4 | 25.00 |

Multiple responses were possible for strategy; percentages therefore do not sum to 100%. Percentages refer to the proportion of participants selecting each option.

- Table S3. Modality and functional scope of use by professional group

**Modality**

| **Profession group** | **Both functions** | **Text (written) only** | **Voice/speech (oral) only** |
|---|---|---|---|
| Administrative staff | 6 (50.0%) | 5 (41.67%) | 1 (8.33%) |
| Nursing staff | 23 (63.89%) | 12 (33.33%) | 1 (2.78%) |
| Medical-technical and other | 58 (81.69%) | 9 (12.68%) | 4 (5.63%) |
| Medical doctors | 60 (63.16%) | 29 (30.53%) | 6 (6.32%) |
| Psychosocial health professionals | 59 (46.46%) | 65 (51.18%) | 3 (2.36%) |
| Other (free text) | 2 (66.67%) | 1 (33.33%) | 0 |

**Functional scope**

| **Profession group** | **Entire conversations** | **Full sentences** | **Specialised single terms** |
|---|---|---|---|
| Administrative staff | 4 (23.53%) | 10 (58.82%) | 4 (23.53%) |
| Medical doctors | 35 (32.11%) | 69 (63.3%) | 37 (33.94%) |
| Medical-technical and other health professions | 40 (54.79%) | 63 (86.3%) | 36 (49.32%) |
| Nursing staff | 14 (31.82%) | 30 (68.18%) | 18 (40.91%) |
| Other (free text) | 1 (25.0%) | 3 (75.0%) | 2 (50.0%) |
| Psychosocial health professions | 20 (11.43%) | 90 (51.43%) | 83 (47.43%) |
| Therapeutic professions | 4 (19.05%) | 6 (28.57%) | 6 (28.57%) |

Modality proportions are calculated based on the sub-sample of participants who actively use tools and provided a modality preference. Multiple responses were possible for functional scope; percentages therefore do not sum to 100%.

- Table S4. Correlation matrix (TAM + contextual variables)

| variable | PEOU | PU | ATT | BI | Encouragement | Conditional Access | Accuracy Concern | Privacy Concern |
|---|---|---|---|---|---|---|---|---|
| Perceived ease of use (PEOU) | 1.0 | 0.67*** | 0.54*** | 0.6*** | 0.13* | 0.09 | -0.1 | -0.08 |
| Perceived usefulness (PU) | 0.67*** | 1.0 | 0.59*** | 0.7*** | 0.14* | 0.23*** | -0.1 | -0.08 |

| | | | | | | | | |
|---|---|---|---|---|---|---|---|---|
| Attitude toward use (ATT) | 0.54*** | 0.59*** | 1.0 | 0.59*** | 0.18** | 0.21*** | -0.07 | -0.02 |
| Behavioural intention (BI) | 0.6*** | 0.7*** | 0.59*** | 1.0 | 0.09 | 0.25*** | -0.06 | -0.07 |
| Encouragement | 0.13* | 0.14* | 0.18** | 0.09 | 1.0 | -0.14* | -0.05 | -0.16* |
| Conditional Access | 0.09 | 0.23*** | 0.21*** | 0.25*** | -0.14* | 1.0 | 0.06 | 0.01 |
| Accuracy Concern | -0.1 | -0.1 | -0.07 | -0.06 | -0.05 | 0.06 | 1.0 | 0.07 |
| Privacy Concern | -0.08 | -0.08 | -0.02 | -0.07 | -0.16* | 0.01 | 0.07 | 1.0 |

Values represent Pearson correlation coefficients. Significance levels are indicated as follows: $p$<0.05 (*), $p$<0.01 (**), $p$<0.001 (***). ATT, attitude toward use; BI, behavioural intention; PEOU, perceived ease of use; PU, perceived usefulness.

- Table S5. Ordinal logistic regression sensitivity analysis for behavioural intention

| **Predictor** | **Log-odds coefficient (*SE*)** | **Odds ratio** | **95% CI** | ***p* value** |
|---|---|---|---|---|
| Perceived ease of use | 0.48 (0.17) | 1.61 | 1.16–2.25 | 0.005 |
| Perceived usefulness | 1.36 (0.20) | 3.89 | 2.63–5.76 | < 0.001 |
| Attitude toward use | 0.69 (0.15) | 2.00 | 1.48–2.71 | < 0.001 |
| Organisational encouragement | -0.12 (0.13) | 0.89 | 0.69–1.14 | 0.361 |
| Conditional access | 0.09 (0.13) | 1.10 | 0.85–1.42 | 0.473 |
| Accuracy concern | 0.05 (0.26) | 1.05 | 0.63–1.76 | 0.844 |
| Privacy concern | -0.23 (0.24) | 0.79 | 0.50–1.27 | 0.339 |
| Age | -0.18 (0.12) | 0.84 | 0.66–1.05 | 0.131 |

Proportional-odds ordinal logistic regression treating the nine observed behavioural intention values as ordered categories; n = 257. PEOU, PU, ATT, organisational encouragement, conditional access, and age were standardised; their odds ratios correspond to a one-standard-deviation increase. Accuracy and privacy concerns were entered as binary indicators (reference: concern not reported). OR > 1 indicates higher odds of reporting a higher behavioural intention category. Log likelihood = −368.00; AIC = 767.99. The ATT estimate should be interpreted cautiously because the reliability of the two-item ATT scale was below the predefined threshold. CI, confidence interval; SE, standard error.

- Table S6. Missing data overview

| **Variable** | **Missing (*n*)** | **Total (*n*)** | **Missing %** |
|---|---|---|---|
| Age | 1 | 258 | 0.4 |
| Behavioural intention | 0 | 258 | 0 |
| Attitude toward use | 0 | 258 | 0 |
| Perceived usefulness | 0 | 258 | 0 |
| Perceived ease of use | 0 | 258 | 0 |
| Encouragement | 0 | 258 | 0 |

| | | | |
|---|---|---|---|
| Conditional Access | 0 | 258 | 0 |
| Accuracy Concern | 0 | 258 | 0 |
| Privacy Concern | 0 | 258 | 0 |
| Work Experience | 0 | 258 | 0 |
| Profession | 0 | 258 | 0 |
| Gender | 0 | 258 | 0 |

- Table S7. Internal consistency of TAM constructs

| | Number of items | Cronbach's α | Inter-item correlation (*r*) | Spearman-Brown coefficient |
|---|---|---|---|---|
| **Total** | | | | |
| Perceived usefulness | 5 | 0.82 | - | - |
| Perceived ease of use | 4 | 0.85 | - | - |
| Attitude toward use | 2 | - | 0.45 | 0.62 |
| Behavioural intention | 2 | - | 0.74 | 0.85 |
| **German sample** | | | | |
| Perceived usefulness | 5 | 0.78 | - | - |
| Perceived ease of use | 4 | 0.78 | - | - |
| Attitude toward use | 2 | - | 0.43 | 0.61 |
| Behavioural intention | 2 | - | 0.72 | 0.84 |
| **Romanian sample** | | | | |
| Perceived usefulness | 5 | 0.87 | - | - |
| Perceived ease of use | 4 | 0.89 | - | - |
| Attitude toward use | 2 | - | 0.48 | 0.65 |
| Behavioural intention | 2 | - | 0.81 | 0.89 |

Cronbach's α was calculated for multi-item scales (PU, PEOU). For two-item scales (ATT, BI), inter-item correlations and Spearman-Brown coefficients are reported. Values ≥0.70 are typically acceptable for internal consistency; lower values for two-item scales should be interpreted with caution.

- Table S8. Multicollinearity diagnostics (VIF)

| **Variable** | **VIFs** |
|---|---|
| Perceived ease of use | 1.99 |
| Perceived usefulness | 2.20 |
| Attitude toward use | 1.68 |
| Encouragement | 1.10 |
| Conditional Access | 1.15 |
| Accuracy concern | 1.03 |
| Privacy concern | 1.04 |
| Age | 1.05 |

VIFs, Variation inflation factors.

- Table S9. Comparison of survey completers and non-completers using available participant characteristics

| **Characteristic** | **Completers, *n* (%)** | **Non-completers, *n* (%)** | **Test statistic** | ***p* value** | **Cramér's V** |
|---|---|---|---|---|---|
| **Country** | | | $\chi^2(3) = 1.45$ | 0.694 | 0.047 |

| | | | | | |
|---|---|---|---|---|---|
| Germany | 258 (56.0) | 123 (60.0) | | | |
| Romania | 185 (40.1) | 75 (36.6) | | | |
| South Africa | 13 (2.8) | 4 (2.0) | | | |
| Netherlands | 5 (1.1) | 3 (1.5) | | | |
| **Professional group** | | | $\chi^2(6) = 33.85$ | < 0.001 | 0.226 |
| Psychosocial health professions | 180 (39.0) | 79 (38.7) | | | |
| Medical doctors | 115 (24.9) | 36 (17.6) | | | |
| Medical-technical and other health professions | 73 (15.8) | 29 (14.2) | | | |
| Nursing staff | 46 (10.0) | 30 (14.7) | | | |
| Therapeutic professions | 22 (4.8) | 2 (1.0) | | | |
| Administrative staff | 17 (3.7) | 8 (3.9) | | | |
| Other (free text) | 8 (1.7) | 20 (9.8) | | | |
| **Workplace setting** | | | $\chi^2(11) = 37.85$ | < 0.001 | 0.239 |
| Hospital | 122 (26.5) | 43 (21.2) | | | |
| Private practice / practitioner's practice | 113 (24.5) | 45 (22.2) | | | |
| Emergency medical services | 68 (14.8) | 30 (14.8) | | | |
| Mental health facility | 37 (8.0) | 17 (8.4) | | | |
| Community health center | 25 (5.4) | 7 (3.4) | | | |
| Outpatient clinic | 25 (5.4) | 11 (5.4) | | | |
| Education / research setting | 18 (3.9) | 3 (1.5) | | | |
| Public health department | 17 (3.7) | 4 (2.0) | | | |
| Other (free text) | 15 (3.3) | 31 (15.3) | | | |
| Rehabilitation center | 9 (2.0) | 4 (2.0) | | | |
| Nursing home / long-term care facility | 8 (1.7) | 4 (2.0) | | | |
| Ambulatory nursing services | 4 (0.9) | 4 (2.0) | | | |
| **Mental-healthcare setting** | | | $\chi^2(1) = 0.12$ | 0.730 | 0.013 |
| Yes | 200 (43.4) | 91 (44.8) | | | |
| No | 261 (56.6) | 112 (55.2) | | | |

| | | | | | |
|---|---|---|---|---|---|
| **Work experience, years, median (IQR)** | 13 (6–23) | 10 (4–20) | W = 41,399 | 0.029 | — |
| **Exposure to language barriers, %, median (IQR)** | 10 (1–25) | 5 (0–30) | W = 26,579.5 | .112 | — |

Values are n (%) unless otherwise stated. Denominators vary because non-completers discontinued the questionnaire at different stages. Available observations for completers/non-completers were: country, 461/205; professional group, 461/204; workplace setting, 461/203; mental-healthcare setting, 461/203; work experience, 461/201; and exposure to language barriers, 457/128. Percentages are based on available responses for each variable. Pearson chi-square tests were used for categorical variables and Wilcoxon rank-sum tests for continuous or ordinal variables. Cramér's V is reported for categorical comparisons. IQR, interquartile range.

- Table S10. Diagnostic results for the adjusted OLS regression model

| **Diagnostic** | **Result** | **Interpretation** |
|---|---|---|
| Participants at the maximum BI score | 34/258 (13.2%) | Some clustering at the upper end of the BI scale |
| Breusch-Pagan test | $\chi^2(8) = 24.20$; $p = 0.002$ | Evidence of heteroscedasticity |
| Shapiro-Wilk test of residuals | $W = 0.984$; $p = 0.005$ | Modest tail deviations from residual normality |
| Maximum Cook's distance | 0.061 | No observation approached the conventional threshold of 1 |
| Cook's distance > 4/n | 20 observations | Observations exceeding the screening threshold were examined for potential influence |
| Cook's distance > 1 | 0 observations | No highly influential observation according to this threshold |

The distribution of behavioural intention was assessed in the full German sample (N = 258). Residual diagnostics were based on the adjusted OLS model (n = 257), which included PEOU, PU, ATT, organisational encouragement, conditional access, accuracy concern, privacy concern, and age. Because the Breusch-Pagan test indicated heteroscedasticity, HC3 heteroscedasticity-consistent confidence intervals and p values were used for the main inferential reporting. BI, behavioural intention; Q–Q, quantile-quantile. OLS, ordinary least squares.

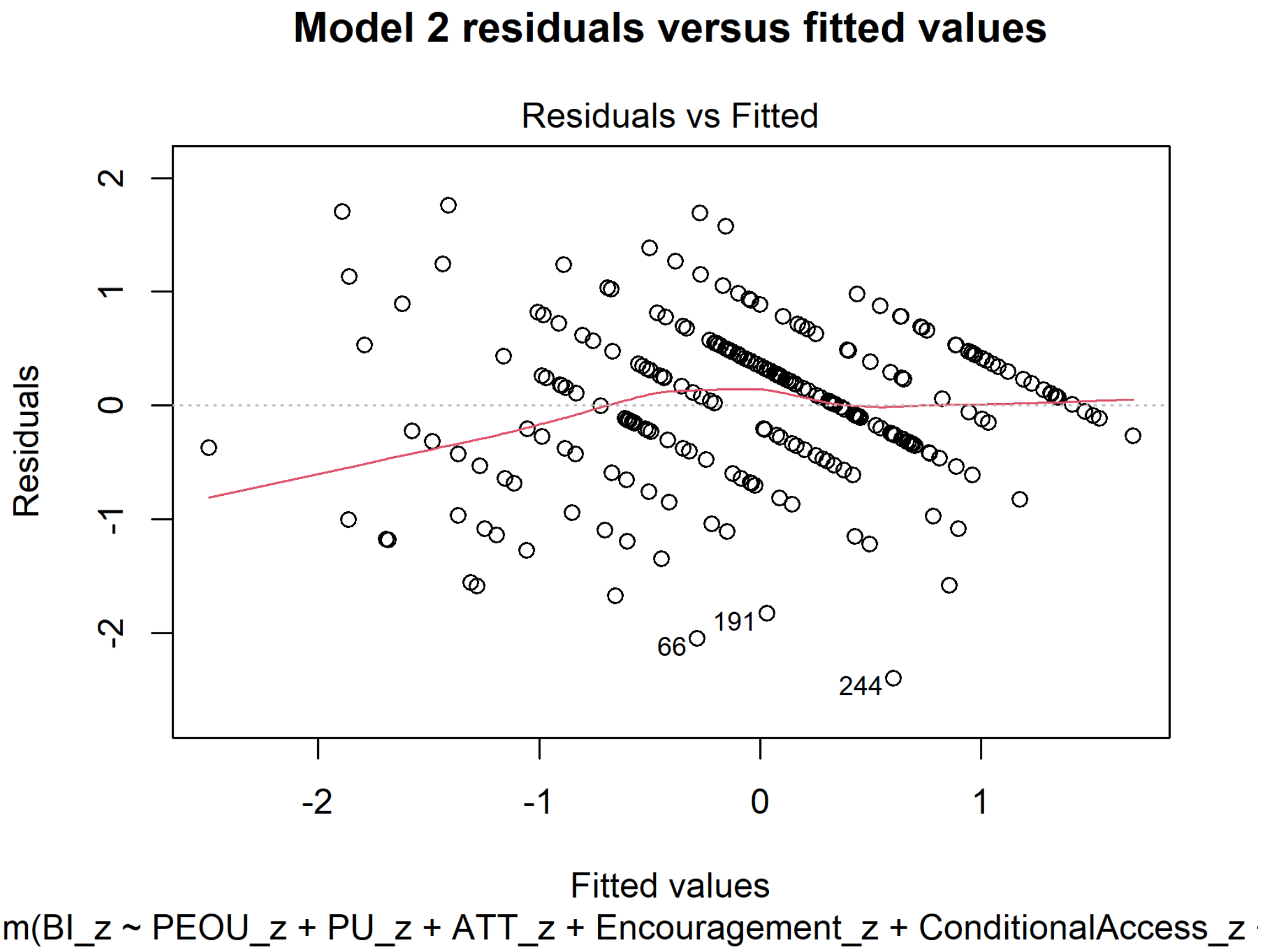


*Figure S 1. Residuals versus fitted values, used to assess the residual pattern and heteroscedasticity*

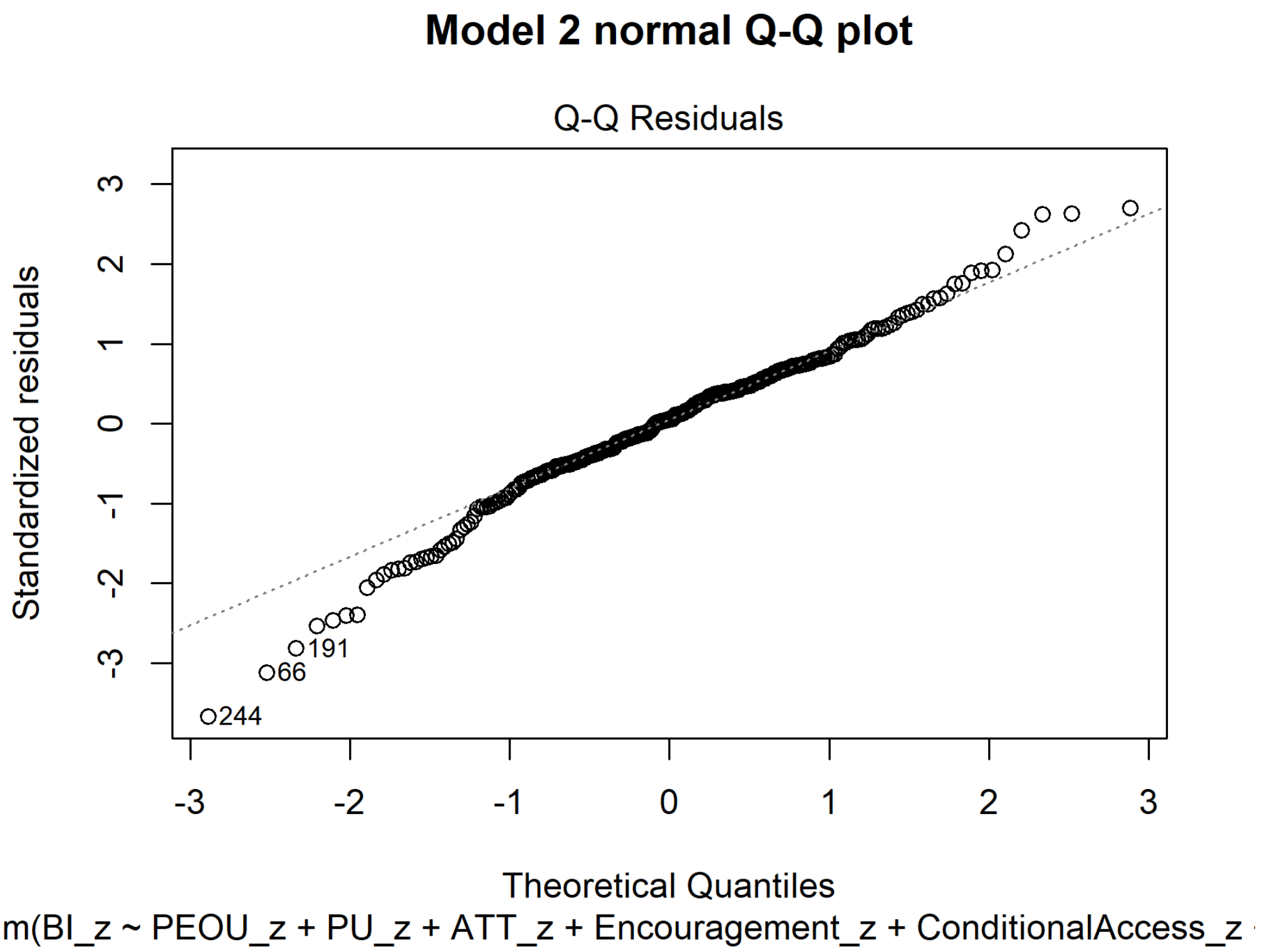


*Figure S 2. Normal Q-Q plot of the model residuals, used to assess departures from residual normality.*

## Model 2 Cook's distance

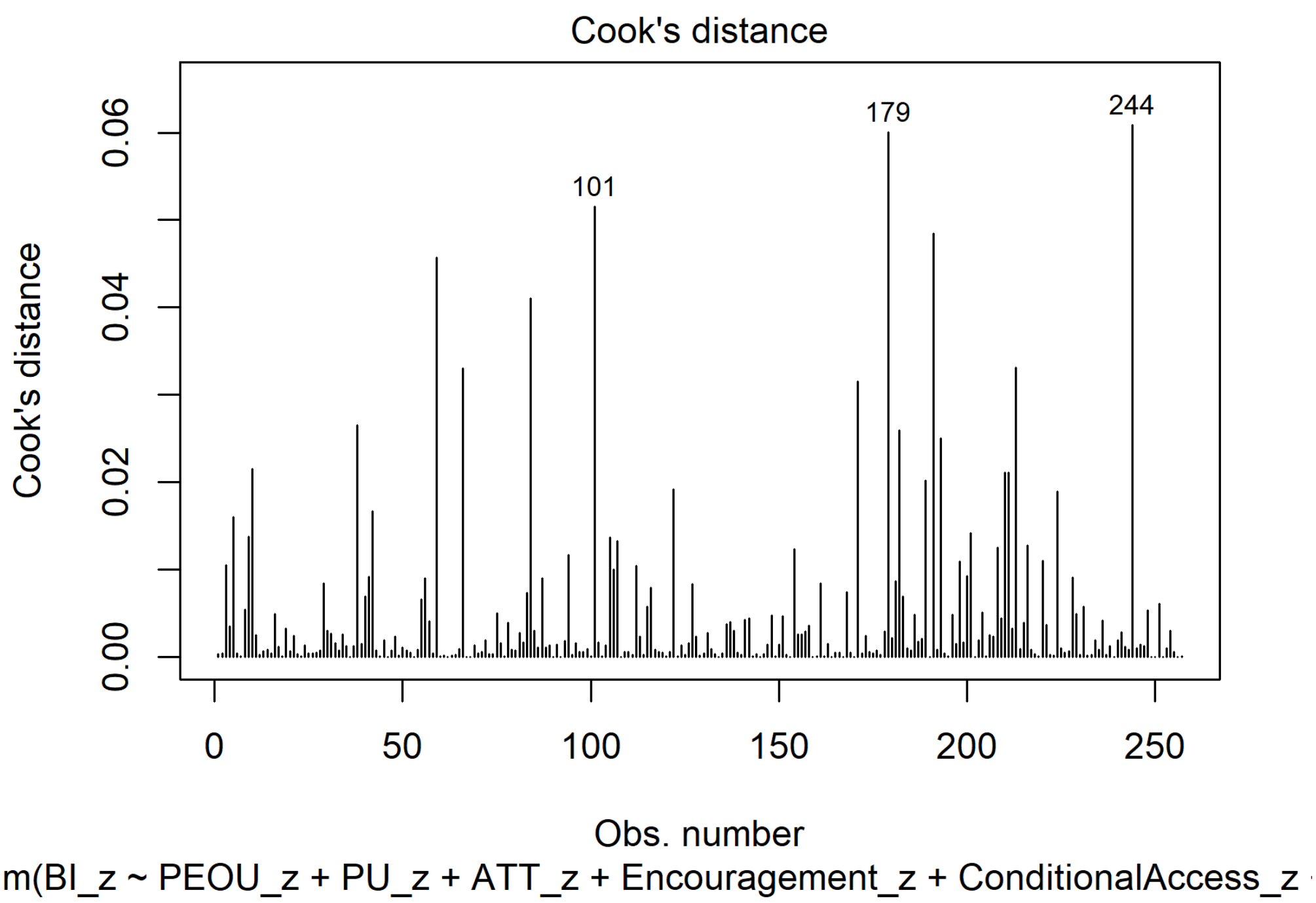


*Figure S 3. Cook's distance for individual observations, used to identify potentially influential cases.*

- Table S11. Sensitivity analyses including exposure to language barriers and expanded covariate adjustment

| **Variable** | **Exposure model β (95% CI)** | ***p* value** | **Expanded model β (95% CI)** | ***p* value** |
|---|---|---|---|---|
| Perceived ease of use | 0.18 (0.04–0.33) | 0.011 | 0.15 (0.0004–0.29) | 0.049 |
| Perceived usefulness | 0.42 (0.25–0.59) | < 0.001 | 0.44 (0.26–0.61) | < 0.001 |
| Attitude toward use | 0.22 (0.11–0.34) | < 0.001 | 0.20 (0.07–0.32) | 0.002 |
| Organisational encouragement | -0.02 (-0.12–0.08) | 0.700 | -0.02 (-0.13–0.08) | 0.643 |
| Conditional access | 0.08 (-0.03–0.18) | 0.141 | 0.04 (-0.06–0.15) | 0.437 |
| Accuracy concern | 0.03 (-0.16–0.22) | 0.779 | 0.02 (-0.17–0.20) | 0.848 |
| Privacy concern | -0.05 (-0.22–0.13) | 0.610 | -0.01 (-0.19–0.17) | 0.883 |
| Age | -0.07 (-0.15–0.006) | 0.072 | -0.06 (-0.26–0.15) | 0.577 |
| Exposure to language barriers | -0.002 (-0.10–0.09) | 0.972 | -0.004 (-0.10–0.09) | 0.943 |
| Work experience | — | — | 0.004 (-0.20–0.20) | 0.972 |
| Mental-healthcare setting: yes | — | — | -0.06 (-0.36–0.24) | 0.692 |
| Gender: male | — | — | -0.11 (-0.33–0.10) | 0.290 |
| Gender: other / prefer not to answer | — | — | -0.29 (-0.94–0.37) | 0.390 |
| **Professional group** | | | | |
| Medical doctors | — | — | 0.46 (0.10–0.82) | 0.012 |
| Nursing staff | — | — | 0.25 (-0.13–0.63) | 0.191 |
| Medical-technical and other health professions | — | — | 0.34 (-0.02–0.71) | 0.065 |
| Therapeutic professions | — | — | 0.43 (-0.12–0.98) | 0.125 |
| Administrative staff | — | — | 0.22 (-0.32–0.76) | 0.416 |

| | | | | |
|---|---|---|---|---|
| Other (free text) | — | — | 0.48 (-1.31–2.28) | 0.597 |
| **Model fit** | | | | |
| *n* | 254 | | 254 | |
| $R^2$ | 0.569 | | 0.604 | |
| Adjusted $R^2$ | 0.553 | | 0.572 | |
| AIC | 528.152 | | 526.318 | |
| BIC | 567.063 | | 600.602 | |

Standardised OLS regression coefficients are reported with HC3 heteroscedasticity-consistent 95% confidence intervals and p values. The exposure model added self-reported exposure to language barriers to the primary adjusted model. The expanded model additionally included work experience, mental-healthcare setting, gender, and professional group. Continuous predictors were standardised. Reference categories were no mental-healthcare setting, female gender, and psychosocial health professions. Accuracy and privacy concerns were entered as binary indicators (reference: concern not reported). ATT estimates should be interpreted cautiously because the reliability of the two-item ATT scale was below the predefined threshold. AIC, Akaike information criterion; BIC, Bayesian information criterion; CI, confidence interval; ATT, attitude toward use; BI, behavioural intention; PEOU, perceived ease of use; PU, perceived usefulness.
