# Supplementary material for: Healthcare providers’ use and acceptance of translation technologies in multilingual healthcare settings: a cross-sectional study
Source: Int J Equity Health. 2026 Oct 21;25:252. doi: 10.1186/s12939-026-03085-6 (PMC13688893; doi:10.1186/s12939-026-03085-6)
Supplement: Supplementary file 3 [file 12939_2026_3085_MOESM3_ESM.pdf]

STROBE Statement—checklist of items that should be included in reports of observational studies

| | Item No. | Recommendation | Page No. | Relevant text from manuscript |
|---|---|---|---|---|
| **Title and abstract** | 1 | (*a*) Indicate the study's design with a commonly used term in the title or the abstract | 1;2 | Title / Abstract |
| | | (*b*) Provide in the abstract an informative and balanced summary of what was done and what was found | 2-3 | Abstract |
| **Introduction** | | | | |
| Background/rationale | 2 | Explain the scientific background and rationale for the investigation being reported | 3-5 | Introduction |
| Objectives | 3 | State specific objectives, including any prespecified hypotheses | 5-6 | Study aims and objectives |
| **Methods** | | | | |
| Study design | 4 | Present key elements of study design early in the paper | 6 | Methods (Study design and setting) |
| Setting | 5 | Describe the setting, locations, and relevant dates, including periods of recruitment, exposure, follow-up, and data collection | 6-7 | Methods (Study design and setting) |
| Participants | 6 | (*a*) *Cohort study*—Give the eligibility criteria, and the sources and methods of selection of participants. Describe methods of follow-up<br>*Case-control study*—Give the eligibility criteria, and the sources and methods of case ascertainment and control selection. Give the rationale for the choice of cases and controls<br>*Cross-sectional study*—Give the eligibility criteria, and the sources and methods of selection of participants | 7 | Methods (participants & recruitment) |
| | | (*b*) *Cohort study*—For matched studies, give matching criteria and number of exposed and unexposed<br>*Case-control study*—For matched studies, give matching criteria and the number of controls per case | | n.a. |
| Variables | 7 | Clearly define all outcomes, exposures, predictors, potential confounders, and effect modifiers. Give diagnostic criteria, if applicable | 9-10 | Methods (Measures and variables); Supplementary file 1 (Variable operationalisation and mapping) |
| Data sources/ measurement | 8* | For each variable of interest, give sources of data and details of methods of assessment (measurement). Describe comparability of assessment methods if there is more than one group | *8-10* | Methods (Survey instrument; Measures and variables); |

| | | | | |
|---|---|---|---|---|
| | | | | Supplementary file 1 (Variable operationalisation and mapping) |
| Bias | 9 | Describe any efforts to address potential sources of bias | 6-7; 10-14; 38-40 | Methods (Participants and recruitment; Data analysis); Discussion (Strengths and limitations) |
| Study size | 10 | Explain how the study size was arrived at | 7-8 | Methods (Sample size determination) |

Continued on next page

| | | | | |
|---|---|---|---|---|
| Quantitative variables | 11 | Explain how quantitative variables were handled in the analyses. If applicable, describe which groupings were chosen and why | 9-14 | Methods (Measures and variables; Data analysis); Supplementary file 1 (Analysis plan and variable operationalisation) |
| Statistical methods | 12 | (*a*) Describe all statistical methods, including those used to control for confounding | 10-14 | Methods (Data analysis: missing data, descriptive and group comparisons, regression models, covariate selection, diagnostics, and sensitivity analyses); Supplementary file 1 (Analysis plan). |
| | | (*b*) Describe any methods used to examine subgroups and interactions | 12 | Methods (Group comparisons and preparatory analyses): exploratory comparisons by professional group and gender. Interaction analyses were not conducted. |
| | | (*c*) Explain how missing data were addressed | 10-11 | Methods (Data preparation and missing data); Supplementary file 1 (Analysis plan) |
| | | (*d*) *Cohort study*—If applicable, explain how loss to follow-up was addressed<br>*Case-control study*—If applicable, explain how matching of cases and controls was addressed<br>*Cross-sectional study*—If applicable, describe analytical methods taking account of sampling strategy | 7 | Methods (Participants and recruitment): convenience and snowball sampling. Sampling weights or design-based adjustments were not applied. |
| | | (*e*) Describe any sensitivity analyses | 12-14; 28-29 | Methods (Regression analyses); Results (Sensitivity analyses); Supplementary file 1 (Analysis plan); Supplementary file 2 (Tables S5 and S11) |
| **Results** | | | | |
| Participants | 13* | (a) Report numbers of individuals at each stage of study—eg numbers potentially eligible, examined | 14-15 | Results (Participant flow and |

| | | | | |
|---|---|---|---|---|
| | | for eligibility, confirmed eligible, included in the study, completing follow-up, and analysed | | attrition analysis; Sample characteristics) |
| | | (b) Give reasons for non-participation at each stage | 14-15 | Results (Participant flow and attrition analysis): numbers completing and discontinuing the survey and reasons for exclusion from the analytic sample. Reasons for individual survey discontinuation were not collected. |
| | | (c) Consider use of a flow diagram | 14 | Results (Participant flow and attrition analysis): participant numbers and exclusions reported in text; no flow diagram included. |
| Descriptive data | 14* | (a) Give characteristics of study participants (eg demographic, clinical, social) and information on exposures and potential confounders | 15-17 | Results (Sample characteristics; Exposure to language barriers), Tables 1 and 2. |
| | | (b) Indicate number of participants with missing data for each variable of interest | 10-11 | Methods (Data preparation and missing data); Supplementary file 2 (Table S6: missingness among German regression variables) |
| | | (c) *Cohort study*—Summarise follow-up time (eg, average and total amount) | | n.a. |
| Outcome data | 15* | *Cohort study*—Report numbers of outcome events or summary measures over time | | *n.a.* |
| | | *Case-control study*—Report numbers in each exposure category, or summary measures of exposure | | *n.a.* |
| | | *Cross-sectional study*—Report numbers of outcome events or summary measures | *19-29* | Results (Use of strategies to overcome language barriers; Acceptance of translation technologies; Multivariable regression; Sensitivity analyses), Tables 3, 7 and 8. |
| Main results | 16 | (*a*) Give unadjusted estimates and, if applicable, confounder-adjusted estimates and their precision (eg, 95% confidence interval). Make clear which confounders were adjusted for and why they were | 25-28 | Results (Preparatory analyses and bivariate associations; |

| | | |
|---|---|---|
| | included | Multivariable regression), Table 8; Supplementary file 2 (Table S4). Covariate selection is explained in Methods (Regression analyses). |
| | (*b*) Report category boundaries when continuous variables were categorized | n.a. |
| | (*c*) If relevant, consider translating estimates of relative risk into absolute risk for a meaningful time period | n.a. |

Continued on next page

| | | | | |
|---|---|---|---|---|
| Other analyses | 17 | Report other analyses done—eg analyses of subgroups and interactions, and sensitivity analyses | 24-25; 28-29 | Results (Acceptance of translation technologies; Sensitivity analyses); Supplementary file 2 (Tables S5 and S11) |
| **Discussion** | | | | |
| Key results | 18 | Summarise key results with reference to study objectives | 29-30 | Discussion (summary of main findings) |
| Limitations | 19 | Discuss limitations of the study, taking into account sources of potential bias or imprecision. Discuss both direction and magnitude of any potential bias | 38-40 | Discussion (Strengths and limitations) |
| Interpretation | 20 | Give a cautious overall interpretation of results considering objectives, limitations, multiplicity of analyses, results from similar studies, and other relevant evidence | 29-42 | Discussion (interpretation of main findings in light of the study design, exploratory analyses, limitations, and previous research) |
| Generalisability | 21 | Discuss the generalisability (external validity) of the study results | 40 | Discussion (Strengths and limitations) |
| **Other information** | | | | |
| Funding | 22 | Give the source of funding and the role of the funders for the present study and, if applicable, for the original study on which the present article is based | 44 | Declarations (Funding and Acknowledgements): funding source and grant ID. The funder had no role in the study design, data collection, analysis, interpretation, or preparation of the manuscript. |

*Give information separately for cases and controls in case-control studies and, if applicable, for exposed and unexposed groups in cohort and cross-sectional studies.

**Note:** An Explanation and Elaboration article discusses each checklist item and gives methodological background and published examples of transparent reporting. The STROBE checklist is best used in conjunction with this article (freely available on the Web sites of PLoS Medicine at http://www.plosmedicine.org/, Annals of Internal Medicine at http://www.annals.org/, and Epidemiology at http://www.epidem.com/). Information on the STROBE Initiative is available at www.strobe-statement.org.
